# Comammox *Nitrospira* among dominant ammonia oxidizers within aquarium biofilter microbial communities

**DOI:** 10.1128/aem.00104-24

**Published:** 2024-06-20

**Authors:** Michelle M. McKnight, Josh D. Neufeld

**Affiliations:** 1 Department of Biology, University of Waterloo, Waterloo, Ontario, Canada; University of Michigan, Ann Arbor, Michigan, USA

**Keywords:** aquarium, biofilters, archaea, comammox *Nitrospira*, nitrification, ammonia oxidation

## Abstract

Nitrification by aquarium biofilters transforms ammonia waste (NH_3_/NH_4_^+^) to less toxic nitrate (NO_3_^-^) via nitrite (NO_2_^-^). Prior to the discovery of complete ammonia-oxidizing (“comammox” or CMX) *Nitrospira*, previous research revealed that ammonia-oxidizing archaea (AOA) dominated over ammonia-oxidizing bacteria (AOB) in freshwater aquarium biofilters. Here, we profiled aquarium biofilter microbial communities and quantified the abundance of all three known ammonia oxidizers using 16S rRNA gene sequencing and quantitative PCR (qPCR), respectively. Biofilter and water samples were each collected from representative residential and commercial freshwater and saltwater aquaria. Distinct biofilter microbial communities were associated with freshwater and saltwater biofilters. Comammox *Nitrospira amoA* genes were detected in all 38 freshwater biofilter samples (average CMX *amoA* genes: 2.2 × 10^3^ ± 1.5 × 10^3^ copies/ng) and dominant in 30, whereas AOA were present in 35 freshwater biofilter samples (average AOA *amoA* genes: 1.1 × 10^3^ ± 2.7 × 10^3^ copies/ng) and only dominant in 7 of them. The AOB were at relatively low abundance within biofilters (average of 3.2 × 10^1^ ± 1.1 × 10^2^ copies of AOB *amoA* genes/ng of DNA), except for the aquarium with the highest ammonia concentration. For saltwater biofilters, AOA or AOB were differentially abundant, with no comammox *Nitrospira* detected. Additional sequencing of *Nitrospira amoA* genes revealed differential distributions, suggesting niche adaptation based on water chemistry (e.g., ammonia, carbonate hardness, and alkalinity). Network analysis of freshwater microbial communities demonstrated positive correlations between nitrifiers and heterotrophs, suggesting metabolic and ecological interactions within biofilters. These results demonstrate that comammox *Nitrospira* plays a previously overlooked, but important role in home aquarium biofilter nitrification.

**IMPORTANCE:**

Nitrification is a crucial process that converts toxic ammonia waste into less harmful nitrate that occurs in aquarium biofilters. Prior research found that ammonia-oxidizing archaea (AOA) were dominant over ammonia-oxidizing bacteria (AOB) in freshwater aquarium biofilters. Our study profiled microbial communities of aquarium biofilters and quantified the abundance of all currently known groups of aerobic ammonia oxidizers. The findings reveal that complete ammonia-oxidizing (comammox) *Nitrospira* were present in all freshwater aquarium biofilter samples in high abundance, challenging our previous understanding of aquarium nitrification. We also highlight niche adaptation of ammonia oxidizers based on salinity. The network analysis of freshwater biofilter microbial communities revealed significant positive correlations among nitrifiers and other community members, suggesting intricate interactions within biofilter communities. Overall, this study expands our understanding of nitrification in aquarium biofilters, emphasizes the role of comammox *Nitrospira*, and highlights the value of aquaria as microcosms for studying nitrifier ecology.

## INTRODUCTION

Aquaria represent artificial environments for housing aquatic life, such as fish and plants, with residential and commercial applications. Nitrogenous waste in the form of ammonia/ammonium (NH_3_/NH_4_^+^), excreted by fish or produced from organic matter degradation, can reach concentrations that are toxic to aquatic life, especially in newly established aquaria. Concentrations of unionized ammonia (NH_3_) greater than 0.1 mg/L can lead to chronic stress and disease for aquarium fish if not removed through nitrification or water changes (1, 2). To maintain a healthy aquarium, biofilters with established microbial communities that can perform nitrification are used to convert ammonia into nitrate, via nitrite, although this intermediate can also lead to toxicity, which reinforces the importance of establishing nitrification rapidly within biofilters to ensure aquatic health.

The first step of nitrification, ammonia oxidation, can be performed by either ammonia-oxidizing bacteria (AOB; e.g., *Nitrosomonas*) or ammonia-oxidizing archaea (AOA; e.g., *Nitrosotenuis*, *Nitrosopumilus*) (3). Nitrite oxidation is subsequently performed by nitrite-oxidizing bacteria (NOB; e.g., *Nitrobacter*, *Nitrospira*). More recently, specific *Nitrospira* spp. were found to catalyze complete ammonia oxidation (“comammox”), possessing genes for both ammonia and nitrite oxidation. Comammox *Nitrospira* were first discovered in a deep oil exploration well and a recirculating aquaculture system (RAS) (4, 5) and were earlier predicted to be associated with biofilm growth exposed to low ammonia concentrations (6). All known comammox bacteria belong to the genus *Nitrospira*, previously only considered to contain canonical NOB (7), and are classified into two distinct clades, comammox clade A and clade B (4). Since their discovery, comammox *Nitrospira* has been identified in many natural and engineered environments, including wastewater and drinking water treatment plants, soil, lake sediments, and groundwater-fed rapid sand filters (8–13). Although factors that govern environmental distributions and abundances of comammox *Nitrospira* remain unclear, several studies demonstrate that comammox *Nitrospira* associated with relatively low ammonia concentrations (9, 12). Kinetic studies reveal that the cultivated comammox representative *Nitrospira inopinata* has a high affinity for ammonia (K_m(app)_ = 63 ± 10 nM NH_3_), suggesting a competitive advantage within oligotrophic environments (14).

Initial aquarium filter studies concluded that AOB were solely responsible for ammonia oxidation in these systems (15, 16). Similarly, NOB were identified as being responsible for completing nitrification in association with AOB, with *Nitrospira* spp. serving as strict nitrite oxidizers (17). However, the first cultivated AOA representative, *Nitrosopumilus maritimus* SCM1, was isolated from gravel collected from a marine tropical aquarium (18), and several studies in subsequent years revealed a dominance of AOA in marine aquaculture biofilters (19, 20). A subsequent survey of ammonia oxidizers within biofilters collected from hobbyist freshwater aquaria, using qPCR analysis of the ammonia monooxygenase A gene (*amoA*), demonstrated that AOA were numerically dominant in freshwater aquaria compared with their AOB counterparts (21). This survey also found that the relative abundance of AOA was negatively correlated with total ammonia concentration. Another study identified a dominance of AOA in freshwater aquarium biofilters, observing long-term stability within the sampled filter material (22). Given the identification of comammox *Nitrospira* in aquaculture systems (23), and the favorable high surface area for biofilm growth and low ammonia environment predicted for optimal growth of comammox *Nitrospira*, we hypothesized that comammox *Nitrospira* would be relatively abundant with aquarium biofilters.

Here, we revisited microorganisms involved in aquarium nitrification, with an emphasis on ammonia oxidizers, and determined the abundance of AOA, AOB, and comammox *Nitrospira* present in representative freshwater and marine aquarium biofilters using qPCR analysis of the *amoA* gene, along with subsequent sequencing of *amoA* genes from comammox *Nitrospira*. We also explored total microbial community composition using 16S rRNA gene sequencing and evaluated aquarium characteristics that may influence microbial community composition. Overall, we demonstrate that both comammox *Nitrospira* and AOA dominate nitrifier communities within freshwater aquarium biofilters.

## RESULTS

### Aquarium samples

Aquarium biofilter and water samples (Table S1) were collected from 38 freshwater aquaria (FW-01 to FW-38) and eight saltwater aquaria (SW-01 to SW-08). Samples were collected from residences and retail pet stores in the Region of Waterloo and the city of Mississauga in Southwestern Ontario, Canada. Approximately half of the sampled aquaria contained live plants, and the average number of fish across all aquaria was ~22, ranging from 0 to 150. Several aquaria were populated with cichlids, algae eaters (e.g., “Plecos”), or guppies, and many contained mixed tropical or marine fish; one aquarium housed a turtle. Aquarium ages averaged 3 years, ranging from 1 month to 13 years, and were subject to diverse maintenance routines (Table S1). Water used for most aquaria was sourced from municipal, reverse osmosis, distilled, or bottled water, with water changes occurring at frequencies ranging from once a week to once every 8 weeks. For most aquarium biofilters, the sponge or floss material was rarely or never replaced. However, several owners replaced sponge/floss material as often as once a week to a frequency of every 2 years of use. At the time of aquarium establishment, five of the freshwater biofilters and one saltwater biofilter were inoculated with aquarium supplements, and within 6 months prior to sampling, only one freshwater aquarium and two saltwater aquaria had been exposed to antibiotics (Table S1). Specifically, FW-11 was treated with Levamisole-HCl and ICH-X (Malachite Green), and both SW-05 and SW-06 were treated with cupramine and erythromycin. Sampled aquaria had a wide range of conditions for size (5–280 gallons), temperature (19.3-28.8°C), alkalinity (0–14 meq/L), pH (6.2–9.3), general hardness (1–52 dGH), and carbonate hardness (1–38 dKH) (Table S2). Measured concentrations of total ammonia were relatively low overall, with an average concentration of ~59 mg/L NH_3_-N. Nitrite was usually undetected or low in most aquaria; only three aquaria had concentrations >1 mg/L NO_2_^-^-N. In contrast, nitrate concentrations were high in most aquaria (>1 mg/L NO_3_^-^-N), with an average concentration of 16.7 mg/L NO_3_^-^-N, with the lowest and highest measured concentrations of 15.3 mg/L NO_3_^-^-N and 106.7 mg/L NO_3_^-^-N, respectively.

### Aquarium biofilter microbial communities

We identified 12,241 unique amplicon sequence variants (ASVs) from the 16S rRNA gene sequence data for all biofilter samples, with a total of 969,251 reads and an average of 21,071 reads per sample. Positive sequencing controls contained expected ASVs of both *Aliivibrio fischeri* and *Thermus thermophilus* at relative abundances of ~60% and ~40% in both replicates, respectively. Both *Proteobacteria* and *Bacteroidota* dominated phylum-level ASV affiliations for all fresh and saltwater samples (Fig. S1). Freshwater biofilter samples had an average relative abundance of 40.1% ± 9.4% for *Proteobacteria* and 23.7% ± 9.0% for *Bacteroidota*, whereas saltwater biofilters had an average relative abundance of 46.3% ± 6.3% and 30.4% ± 9.5% for the two phyla, respectively. Members of the *Planctomycetota* were also detected consistently across all samples, ranging between a relative abundance of 1% and 13%. Several additional phyla were present in most samples at >1% relative abundance, including *Acidobacteria*, *Chloroflexi*, *Cyanobacteria*, *Myxococcota*, *Nitrospirota*, and *Verrucomicrobiota* (Fig. S1).

**Fig 1:**
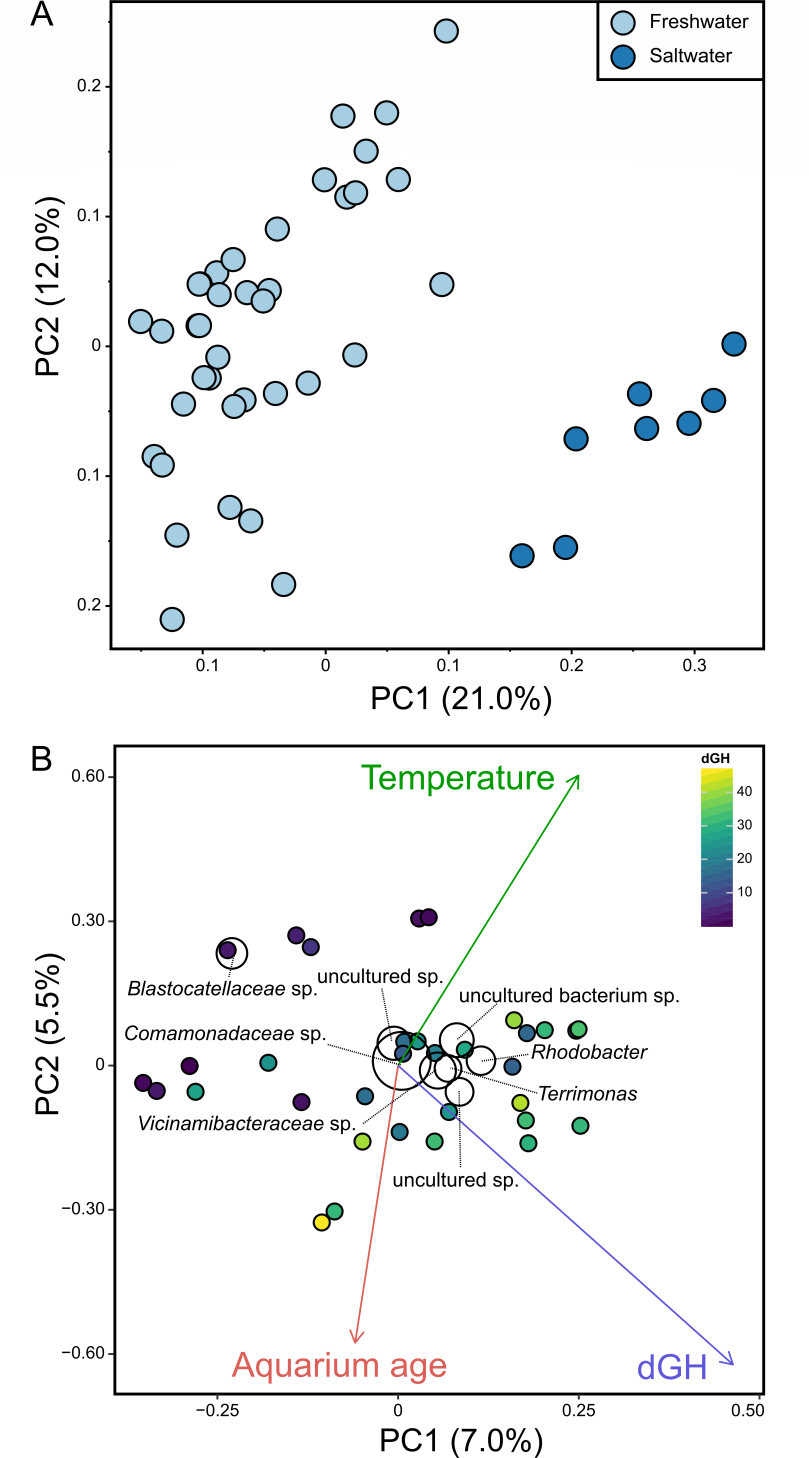
Principal coordinates and triplot analysis of 16S rRNA gene profiles of aquarium biofilters (**A, B**). Grouping between the saltwater and freshwater samples is shown in the PCoA plot (**A**), which was calculated using the weighted UniFrac metric on samples rarefied to the lowest sample count. An ordination based on the Bray-Curtis metric using only the freshwater samples (**B**) shows that the freshwater biofilm communities are correlated with temperature, aquarium age, and dGH (*R*^2^ > 0.3, *P* < 0.05). Taxa collapsed at the phylum level present at an average abundance of greater than 1% are displayed on the plot by the labeled black circles. The size of the circles illustrates the average relative abundance of the species across samples, whereas their placement reflects the correlation specific species have with different samples. The axes on both plots display the percent variation within the samples illustrated by each of the two principal components displayed (**A, B**).

Microbial community profiles of saltwater and freshwater samples were unique, grouping distinctly from one another in multidimensional space (Fig. 1A, pseudo-*F* = 9.6, *P* = 0.001). Although salinity explained the overall composition of sampled aquarium biofilters, other measured parameters also affected microbial community composition. Within freshwater aquarium biofilter samples specifically, correlation analysis revealed that aquarium age, general water hardness, and aquarium temperature all correlated significantly with 16S rRNA gene profiles (*R*^2^ > 0.3, *P* < 0.05; Fig. 1B). For freshwater samples, several taxa grouped in the center of ordination space, indicating that they were common to most freshwater biofilter samples, including ASVs affiliated with *Comamonadaceae*, *Vicinamibacteraceae*, *Rhodobacter*, and *Terrimonas*. In contrast, *Blastocatellaceae*-associated taxa grouped with samples that had lower general hardness (Fig. 1B), indicating that these bacteria were at higher relative abundances in soft water. When considering saltwater samples specifically, only aquarium size correlated with an overall distribution of microbial community profiles (*R*^2^ > 0.3, *P* < 0.05), and this was also true for the combined analysis with salt and freshwater samples. However, the majority of saltwater biofilter samples were collected from the same retail location, where the aquaria were relatively large compared with most at-home aquaria (170–280 gallons; Table S2), and the single home saltwater sample had a size of 55 gallons. With a size range of 5–155 gallons, most freshwater aquaria were smaller with 23 of 38 being less than 55 gallons. This difference in size distribution between salt and freshwater aquarium samples collected, along with the observation that they have different overall microbial community compositions, is likely responsible for the correlation between aquarium size and microbial community composition.

**Fig 2:**
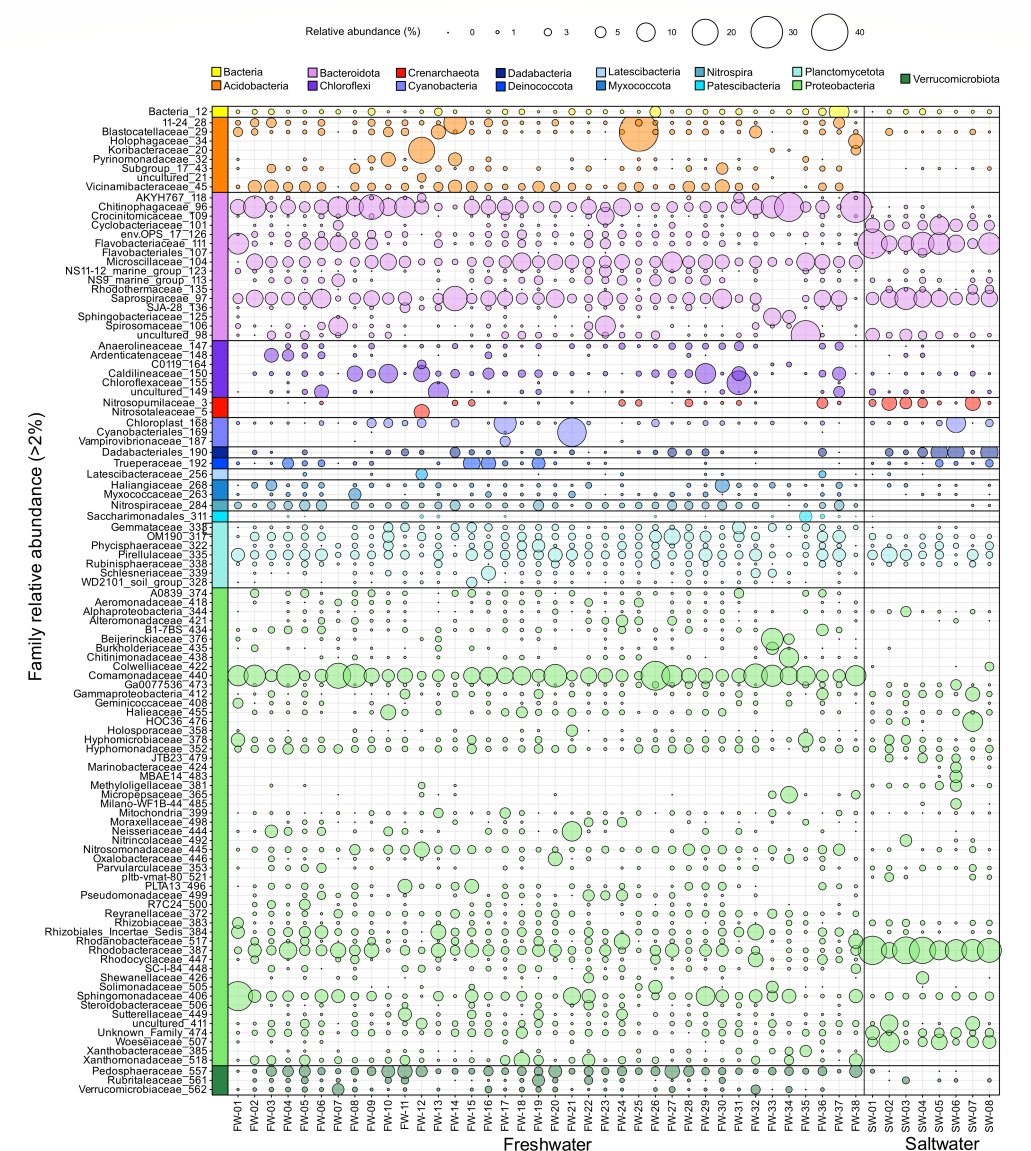
Relative abundance plot of 16S rRNA gene profiles of aquarium biofilter samples at the family level (> 2% RA), grouped and colored by phylum association. Taxonomy has been assigned using the SILVA 138 SSU database, with taxonomy shown either at the family level or at the lowest assigned taxonomic level above family. Taxa present in at least one sample greater than 2% relative abundance are displayed on the plot.

At the taxonomic family level, community profiles were more distinct between saltwater and freshwater biofilter samples (Fig. 2). Members of the families *Saprospiraceae* and *Rhodobacteraceae* were detected in both fresh and saltwater biofilters; *Rhodobacteraceae* had higher relative abundance in saltwater samples (between 7% and 24%) than in freshwater samples (between 2% and 9%). Taxa from families *Cyclobacteraceae* and *Woeseiaceae* taxa appeared only in saltwater biofilter samples at >2% relative abundance thresholds, whereas members of *Chitinophagaceae*, *Microscillaceae*, *Comamonadaceae*, and *Sphingomonadaceae* were present in all freshwater samples either above or below the relative abundance threshold used (Fig. 2). Overall, results from 16S rRNA gene sequencing revealed distinct microbial communities in saltwater and freshwater aquarium biofilters.

### Ammonia oxidizers in aquarium biofilters

Ammonia oxidizer abundance was explored using qPCR targeting *amoA* genes of AOA, AOB, and comammox *Nitrospira*. Additionally, 16S rRNA genes of bacteria and archaea were quantified. An initial screening with primers sets targeting *amoA* genes from two different clades of *Nitrospira,* clade A and clade B (12), revealed that there were no detectable clade B comammox *Nitrospira* present in the aquarium biofilter samples (data not shown). Therefore, only primers associated with clade A comammox *Nitrospira* were used for subsequent qPCR assays.

**Fig 3:**
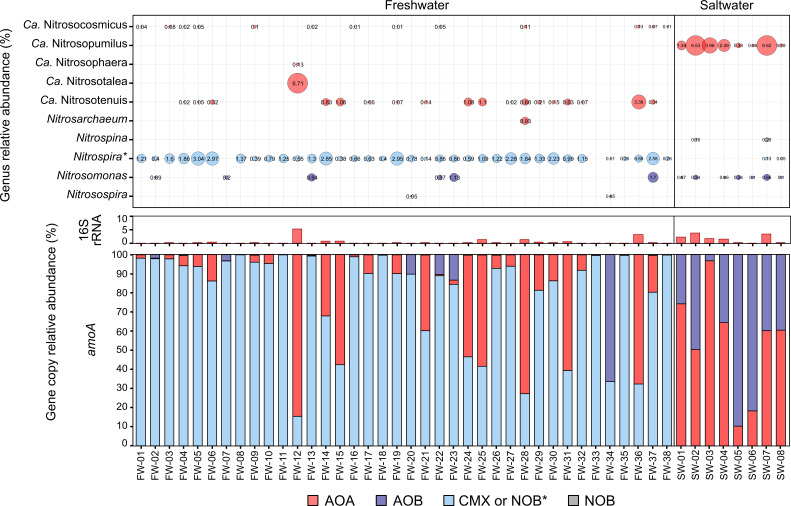
Abundance of microorganisms associated with ammonia oxidation was explored using both 16S rRNA gene profiles and gene abundances of the *amoA* gene, a marker for ammonia oxidation. Relative gene abundance of 16S rRNA genes from the archaeal phylum *Thaumarchaeota* or *Thermoproteota* associated with AOA compared to total 16S rRNA genes (archaeal and bacterial)*,* is shown in the middle. The qPCR results of the relative abundance of *amoA* genes from all three groups of ammonia oxidizers (AOA, AOB, and CMX) detected in biofilter samples are shown with the gene relative abundance of each group determined using the gene copy proportion for a single group relative to the total *amoA* gene copy number per ng of DNA from all three groups (bottom). The genera associated with ammonia or nitrite oxidizing microorganisms present in the aquarium biofilter samples identified from the 16S rRNA gene sequences are illustrated by the bubble plot (top) with the size and number for each bubble representing the relative abundance of each genus in each sample. Colors of bars and bubbles represent the respective groups of ammonia oxidizers, nitrite oxidizers, or comammox.

Clade A comammox *Nitrospira amoA* genes were detected for all 38 freshwater aquarium biofilter samples and were dominant among ammonia oxidizers for 30 of the freshwater biofilters, with *amoA* genes of AOA dominant in the other seven freshwater biofilters (Fig. 3). Overall, the average number of comammox *Nitrospira amoA* gene copies detected in freshwater biofilters at 2.2 × 10^3^ ± 1.5 × 10^3^ copies/ng of DNA was significantly greater than the average concentration of AOA *amoA* genes 1.1 × 10^3^ ± 2.7 × 10^3^ copies/ng (*P* < 0.001). The AOB *amoA* genes had the smallest concentration in the freshwater biofilters, with an average of 3.2 × 10^1^ ± 1.1 × 10^2^ copies/ng of DNA, which was significantly lower than *amoA* concentrations of AOA and CMX (*P* < 0.001). The AOB-specific *amoA* genes occurred at >1% relative abundance (compared with CMX and AOA) in only five other freshwater biofilter samples and dominated in one sample (FW-34), albeit at a concentration of only 6.8 × 10^1^ copies/ng that suggests ammonia oxidizers were not well established in this biofilter (Fig. 3; Table S5). For the eight saltwater biofilters, AOA were the dominant ammonia oxidizers in five biofilters, AOB in two, and one biofilter had similar proportions of AOA and AOB (Fig. 3). The average concentration of AOA *amoA* genes in saltwater biofilters was 2.5 × 10^2^ ± 2.5 × 10^2^ copies/ng of DNA and was not significantly different than the average *amoA* gene concentration for AOB of 2.0 × 10^2^ ± 1.5 × 10^2^ copies/ng.

In addition to *amoA* gene copies, 16S rRNA genes associated with bacteria and archaea were quantified. All aquarium biofilter samples were dominated by bacterial 16S rRNA genes (>94%), whereas 16S rRNA genes associated with archaea were present at lower relative abundances across all samples (Table S5; Fig. 3). The average concentration of bacterial 16S rRNA gene copies detected with qPCR was 3.8 × 10^5^ ± 2.8 × 10^5^ copies/ng of DNA and 2.4 × 10^5^ ± 7.9 × 10^4^ copies/ng of DNA in freshwater and saltwater biofilters, respectively. For the archaeal-associated 16S rRNA genes, there were 1.5 × 10^3^ ± 4.0 × 10^3^ copies/ng of DNA and 3.4 × 10^3^ ± 2.9 × 10^3^ copies/ng of DNA freshwater and saltwater biofilters, respectively. The large standard deviation for archaeal 16S rRNA gene copies in freshwater samples highlights the broad range of copies detected among the freshwater biofilter (Table S5). Highlighting consistency in results between qPCR gene targets, most samples that had a higher relative abundance or concentration of archaeal 16S rRNA genes also had high relative abundances of AOA *amoA* genes (Fig. 3).

For all saltwater samples, *Nitrosopumilus* was the only AOA genus identified. Detected genera of known AOB included *Nitrosomonas*, which were found in all but one sample. The NOB present in saltwater samples included *Nitrospina* and *Nitrospira* genera, the latter of which were likely strict nitrite oxidizers as no comammox *Nitrospira* were detected in the saltwater samples by qPCR amplification. However, not all saltwater samples contained sequences affiliated with known NOB genera (i.e., SW-01, SW-03, SW-04, and SW-05), which might be explained by the short time that the biofilter material was kept (~1 week) for SW-03–05. For the others, it remains possible that NOB might be present below the detectable limits of our 16S rRNA gene sequencing or located elsewhere in the aquarium system. In contrast to the saltwater samples, freshwater samples had a higher diversity of AOA, including *Nitrosocosmicus* and *Nitrosotenuis* spp., which were detected in many of the freshwater biofilters. Additionally, AOA genera *Nitrososphaera, Nitrosotalea*, and *Nitrosoarchaeum* were detected, but each was present in only one sample. Two AOB genera, *Nitrosomonas* and *Nitrosospira*, were detected in several freshwater samples. The genus *Nitrospira* was found in all but two freshwater samples and likely represents both comammox *Nitrospira* and canonical NOB *Nitrospira*, which are indistinguishable using 16S rRNA genes. No other NOB-associated genera were detected in the freshwater samples.

Although the overall relative abundances of ammonia oxidizers detected with 16S rRNA gene sequencing matched well with qPCR relative abundances, there were some discrepancies. For example, samples FW-07 and FW-33 did not have any detectable *Nitrospira* via 16S rRNA gene sequencing but did show detectable comammox *Nitrospira* with *amoA* gene qPCR. Additionally, FW-33 did not have any genera associated with known nitrifiers from 16S rRNA gene data. The lack of detected ASVs associated with nitrifiers for some samples might be explained by their low relative abundance within the community, which might have fallen below detection limits during Illumina library preparation, where bias during PCR amplification and sequencing often favors more abundant organisms in the samples. The sequences of 16S rRNA gene primers used for sequencing are meant to target a broad range of prokaryotes within a sample, whereas the qPCR primers for functional gene detection of *amoA* genes were designed to amplify a more specific sequence, reducing the bias that is seen with the 16S rRNA gene primers.

Because there were several ASVs with taxonomic assignments that could not be resolved below the family level to nitrification-associated taxonomic families, it is possible that these ASVs are poorly characterized nitrifiers or other related taxa with other encoded functions. For example, the *Nitrosomonadaceae* at the family level comprised a greater relative abundance (Fig. 2) than what is seen at the genus level for AOB (Fig. 3). Many of these *Nitrosomonadaceae* ASVs were not assigned to a known AOB genus, and we cannot confirm based on the classification at the 16S rRNA family level that they are AOB. There was also a discrepancy in the relative abundance of *Nitrosomonadaceae* at the family level and the number of AOB detected via qPCR that may suggest they are not all AOB. Although there were some differences between qPCR and 16S rRNA gene sequencing results for AOA and comammox *Nitrospira* abundances, the discrepancy observed in the relative abundance of AOB detected with qPCR and *Nitrosomonadaceae* from the 16S rRNA gene data was consistent in almost all samples.

In addition to identifying dominant ammonia oxidizers present in the biofilters, we explored correlations between ammonia oxidizer abundance and aquarium characteristics. Our results indicated that total ammonia concentration was likely linked to ammonia oxidizer relative abundances. For the samples collected, there was only one aquarium that had a relatively high concentration of total ammonia (FW-34; 651 mg/L NH_3_-N), and this sample also had the highest relative abundance of AOB based on *amoA* gene quantification (Fig. 3). Although as previously noted, the concentration of AOB *amoA* genes detected in this sample was quite low (68.0 copies/ng DNA), although AOB is the dominant ammonia oxidizer in the sample, which suggests that this biofilter has an overall low abundance of ammonia oxidizers present. The majority of other freshwater biofilters contained ammonia oxidizing genes at concentrations 10 to 100 times greater than what is found in FW-34. As for AOB in all other freshwater samples, they fell below 14% relative abundance, with most AOB *amoA* genes making up <1% of the total ammonia oxidizers detected with qPCR. Despite AOB being the dominant ammonia oxidizer in FW-34, this sample has a low overall abundance of both comammox *Nitrospira* and AOB when examining the overall microbial community based on 16S rRNA gene sequencing, where associated genera of *Nitrospira* and *Nitrosomonas* are both present at a relative abundance of <0.05%, suggesting that it may have a poorly established nitrifier community.

### Clade A comammox *Nitrospira* in freshwater biofilters

**Fig 4:**
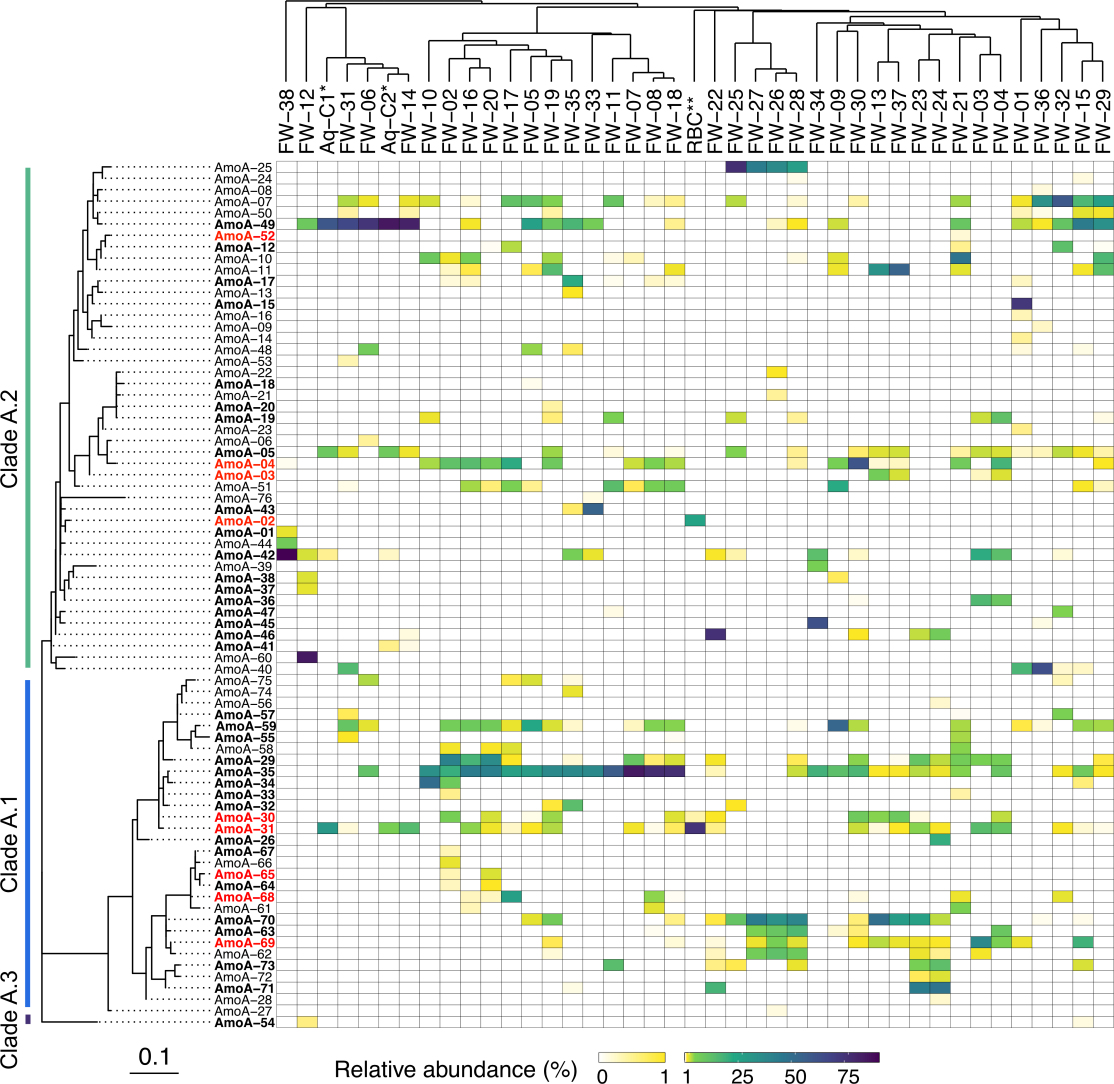
Heatmap shows the relative abundance of different clade A comammox *Nitrospira* detected across freshwater aquarium biofilter samples based on AmoA (ammonia monooxygenase A) amino acid sequences. Biofilter samples were sorted using hierarchical clustering based on sample profile similarity. A maximum likelihood tree displays the relation of AmoA sequences detected within biofilter samples with the Le Gascuel evolutionary model and discrete Gamma distribution to model evolutionary rate differences among sites (Le and Gascuel 2008). An additional positive control sample (RBC**) was included and originates from a wastewater treatment plant, and samples from enrichment cultures inoculated from biofilter FW-14 (Aq-C1* and Aq-C2*) are also shown on the heatmap. Bolded AmoA sequences indicate amino acid sequences already present in the NCBI NR database. AmoA sequences highlighted in red are those that are identical to clade A comammox *Nitrospira* sp. either associated with cultured members or those represented by metagenome bins.

To further explore the distribution of comammox *Nitrospira* within freshwater biofilters, we performed amplicon sequencing of *amoA* genes from clade A comammox *Nitrospira* for all freshwater samples. Final processed *amoA* gene sequence data yielded 248 unique ASVs and 76 unique translated AmoA amino acid sequences (Table S6). Approximately half (i.e., 43) of these sequences shared 100% identity to the NCBI RefSeq non-redundant protein database, including several previously cultured comammox *Nitrospira* species (Table S7). The remaining 33 AmoA sequences were unique to this study. In most freshwater samples, one detected AmoA sequence dominated compared with the other sequences detected (Fig. 4). More than half of the identified AmoA sequences were grouped into clade A.2, whereas most of the remaining sequences were grouped with sequences previously classified into clade A.1. A single AmoA sequence grouped as a third sub-clade, likely corresponding to clade A.3, and was present only at <1% relative abundance in two of the freshwater biofilters. One of the dominant sequences that grouped within clade A.1, found in most samples, was AmoA-35 (Fig. 4), whose sequence was previously identified in a wetland environment (Table S7). Among sequences from clade A.2, AmoA-49 appeared predominantly in many samples (Fig. 4). This sequence was previously found in agricultural soil samples (Table S7).

Samples containing DNA from aquarium enrichment cultures Aq-C1 and Aq-C2, originally inoculated from the FW-14 aquarium biofilter, were also included for *amoA* sequencing (Fig. 4). The goal of the enrichment culture was to experimentally demonstrate the nitrification activity of the biofilm material by enriching nitrifiers, including the same comammox *Nitrospira* sp. that were detected in the biofilter of origin. However, the differential contribution of nitrifiers in the aquarium enrichment (including AOA, NOB, and comammox) was not assessed here. Four AmoA sequences were detected in both enrichment culture samples, with three belonging to clade A.2 and the other belonging to clade A.1. These AmoA sequences were detected within the original inoculum filter sample (FW-14) and in many of the other sampled freshwater biofilters. An additional sample from a Guelph, Ontario wastewater treatment plant rotating biological contactor (RBC) enrichment culture, already known to contain several strains of clade A comammox *Nitrospira*, was also included during sequencing. The comammox *Nitrospira* AmoA amino acid sequences identified in the RBC sample matched with previous *amoA* gene sequences from the Guelph RBCs (9) and were detected in several of the freshwater biofilter samples (Fig. 4).

**Fig 5:**
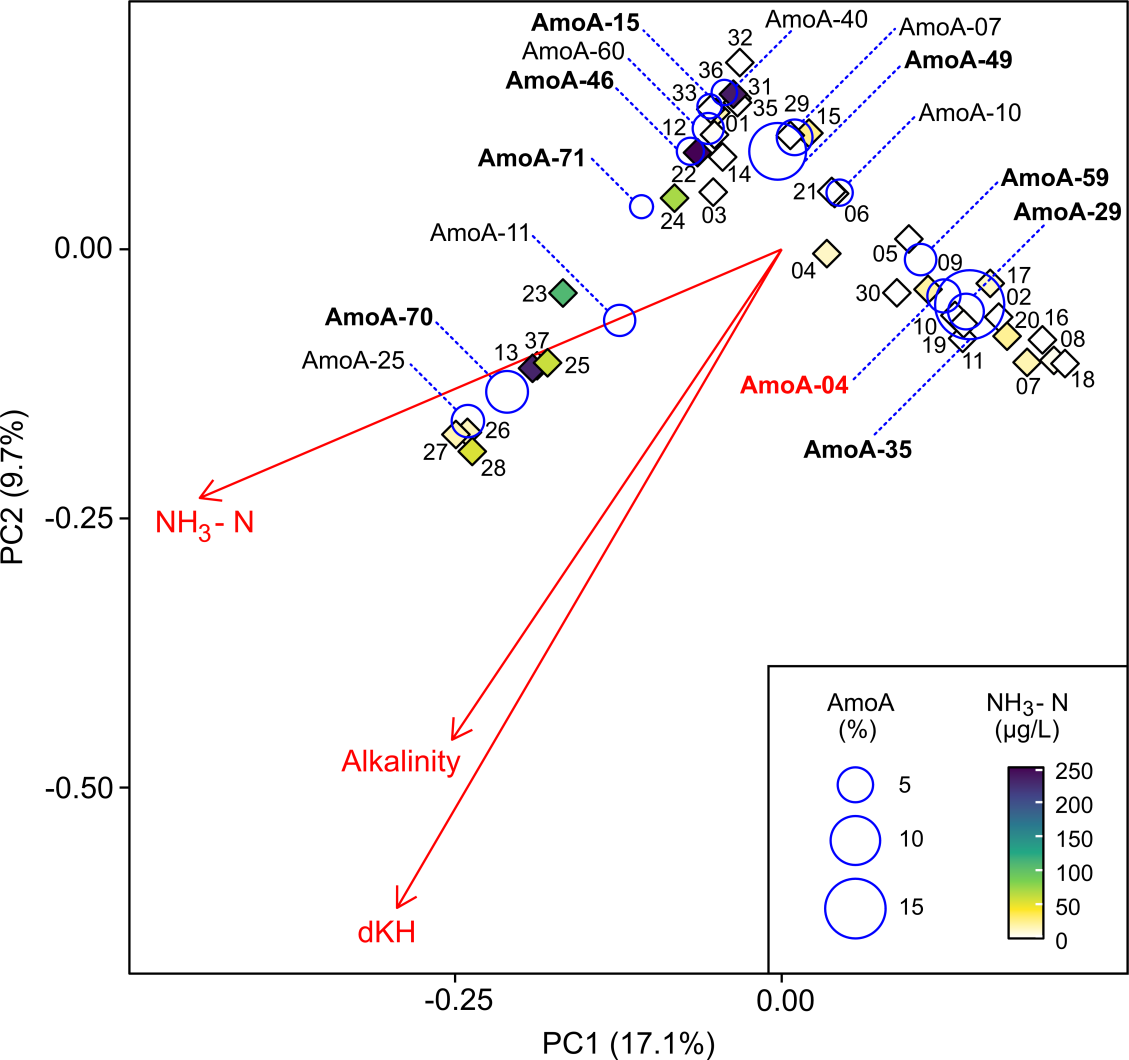
A triplot ordination of clade A *amoA* gene sequences detected in each freshwater biofilter (labeled with their associated FW sample number) based on the Bray-Curtis metric shows significant correlations of detected *amoA* gene sequences with biofilter alkalinity, carbonate hardness, and total ammonia concentration (*R*^2^ > 0.3, *P* < 0.05). Vector arrow size represents the magnitude of the coefficient of determination. AmoA amino acid sequences detected at above 2% as a weighted average abundance across all samples are displayed on the plot by the labeled blue circles. Circle size represents the weighted average relative abundance of the sequence across all samples, whereas their placement reflects the correlation specific species have with different samples. Ordination axes display the percent variation within the samples explained by each principal component (PC1 and PC2). Bolded AmoA sequences indicate amino acid sequences already present in the NCBI NR database. AmoA sequences highlighted in red are those that are identical to clade A comammox *Nitrospira* spp. either associated with cultured members or those represented by metagenome bins.

To explore potential factors that may be correlated with comammox *Nitrospira* spp. distributions across all freshwater biofilter samples, ordination analysis was performed using the comammox *Nitrospira amoA* gene amplicon data (Fig. 5). Ammonia concentration, carbonate hardness, and alkalinity were all significantly correlated with comammox *Nitrospira* community composition (*R*^2^ > 0.3, *P* < 0.05; Fig. 5), suggesting possible niche preference of certain comammox *Nitrospira* to different water quality parameters, such as ammonia concentration and alkalinity. Several residence-specific patterns were also apparent (e.g., FW-25 to FW-28), but generally, samples did not group within ordination space based on the location sampled.

### Analysis of microbial community networks involving nitrifying microorganisms

**Fig 6:**
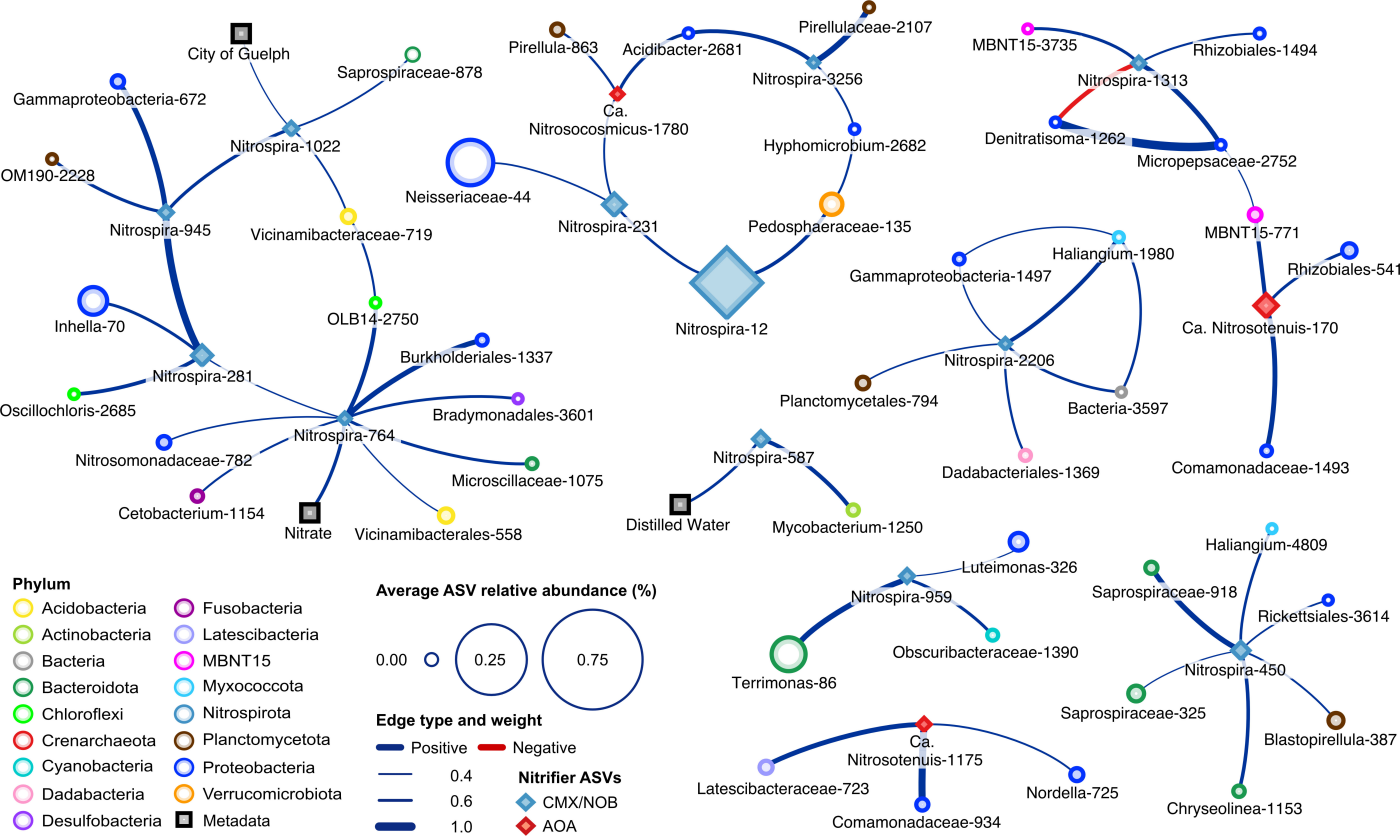
Network analysis displays FlashWeave edge weight values between nitrifier ASVs and other ASVs across freshwater biofilter microbial communities. Each node represents an individual ASV based on 16S rRNA gene sequencing, and network edges represent the FlashWeave edge weight value. Only significant edges associated with nitrifier genera (*P* < 0.05) with a correlation magnitude illustrated by edge width are shown in the network. Color and node shape show the different phyla and associated nitrifier groups, respectively, and node size displays the average relative abundance of the ASV across all 38 freshwater biofilter samples. The lowest taxonomic assignment of each ASV is shown on the node, followed by the individual ASV number ID.

To better understand ammonia oxidizer distributions, we explored correlations among nitrifiers and other taxa within the 16S rRNA gene sequence data using FlashWeave, which infers interactions among microbial populations while incorporating environmental metadata to remove indirect network interactions (24). The interaction network was filtered to show microbial taxa (ASVs) that had significant correlations with known nitrifiers (e.g., AOB, AOA, CMX, and NOB taxa) in the freshwater biofilters (*P* < 0.05, Fig. 6; Fig. S2). The filtered network contained a total of 62 nodes and 59 edges. All edges were positive except for one, which represented mutual exclusions of the connected *Nitrospira* and *Denitratisoma* ASVs. The magnitude of significant edge weights (*P* < 0.05) ranged from 0.33 to 0.99, with an average of 0.49 ± 0.21 (SD). Of the 15 nitrifier nodes in the network, three were associated with AOA genera (e.g., *Nitrosocosmicus*, *Nitrosotenuis* spp.), and 12 nodes were associated with *Nitrospira* genera (Fig. 6). There were no ASVs belonging to AOB genera present in the network. As the FlashWeave analysis also considers metadata, three nodes in the network represented the City of Guelph sampling location, water source (Distilled Water), and nitrate (NO_3_^-^-N). Each of these metadata categories had positive edge connections to distinct *Nitrospira* nodes. The *Nitrospira* and AOA ASVs had comparable levels of connectivity within the network with 4.08 ± 1.93 (SD) edge connections and 3.00 ± 0.00 (SD), respectively. *Nitrospira* ASVs showed higher connectivity to one another, with four edges between neighboring *Nitrospira* nodes. In contrast, only one edge connected an AOA node belonging to a *Nitrosocosmicus* spp. and a *Nitrospira* node. This suggests that the coexistence of several *Nitrospira* spp. may be an important characteristic of their ecology in freshwater aquarium biofilters, but not for AOA and AOB, the latter of which were not present at all within the network.

Only six of the total edges within the network are connected between heterotroph ASVs. Most non-nitrifier ASVs in the network likely represent aerobic heterotrophic bacteria, with several possibly associated with denitrification (e.g., *Denitratisoma*, *Planctomycetota*) and/or nitrogen fixation (e.g., *Rhizobiales, Commamonadaceae*), suggesting further nitrogen cycling beyond nitrification within some of these freshwater biofilters. Despite an inability to distinguish between canonical and comammox *Nitrospira* in the network analysis, the number of positive connecting edges between *Nitrospira* ASV nodes and other putative heterotrophs suggests that their coexistence with *Nitrospira* may be important for nitrifier establishment within biofilter communities (Fig. 6).

## DISCUSSION

Our study provides a new perspective on aquarium ammonia-oxidizers, revealing that most freshwater home aquarium biofilters are dominated by comammox *Nitrospira*. Although previous work showed that AOA were dominant over AOB, prior to the ability to detect comammox counterparts (21), here, we now offer a more complete picture including all three known players. The identification of comammox *Nitrospira*, alongside AOA as dominant ammonia oxidizers in freshwater biofilters, further overturns a decades-old dogma that AOB are responsible for autotrophic ammonia oxidation and *Nitrospira* is responsible for only nitrite oxidation in home aquarium biofilters (15, 17). This could have important implications for the future development of improved aquarium supplement products that are designed to support the establishment of aquarium nitrification, which until now have focused only on the incorporation of AOA, AOB, and NOB in their nitrifying consortia (25, 26). In addition, our results have provided further insight into the ecology of nitrification in aquarium biofilters, which as their own enclosed ecosystems can make excellent analogs for the study of nitrifying communities in natural environments.

### Dominant ammonia oxidizers in aquarium biofilters

Our reassessment of ammonia-oxidizing microorganisms in aquarium biofilters revealed an abundance of comammox *Nitrospira*, revising previous descriptions of known aquarium ammonia oxidizers (21). The ubiquitous presence and observed co-occurrence of multiple comammox *Nitrospira* strains within freshwater aquarium biofilters is consistent with that of similar environments, such as recirculating aquaculture system biofilters, groundwater-fed biofilters, and aquaponics systems, along with wastewater treatment plant (WWTP) rotating biological contactors (9, 13, 23, 27). Within biofilters, the relatively low ammonia concentrations and high surface area for biofilm growth are ideal predicted conditions for microorganisms with slow growth rates and high yields, such as comammox *Nitrospira*, making their presence in freshwater biofilters unsurprising (6). The detection of sole clade A comammox *Nitrospira* in freshwater biofilters reflects current studies, where most comammox *Nitrospira* associated with wastewater treatment systems (e.g., activated sludge and rotating biological contactors) and freshwater environments also belong to clade A *Nitrospira* (9, 28–31). In contrast, clade B comammox *Nitrospira* is identified frequently as the dominant comammox bacteria in forest soils, paddy soils, plateau soils, and river sediments, with experimental confirmation of their active contributions to nitrification in forest soils (29, 32), suggesting some niche preference for between clades for either aquatic or soil environments. However, co-occurrence of clade A and B comammox *Nitrospira* has been observed in metagenomic surveys of terrestrial subsurface samples (33), and clade B was dominant in a groundwater-fed rapid sand filter, existing alongside clade A comammox *Nitrospira* (13). Overall, the underlying factors affecting the distributions of these clades are not entirely understood; however, our results show clade A comammox *Nitrospira* dominates in freshwater aquarium biofilters.

The comammox *Nitrospira* clades A.1 and A.2 detected in our freshwater biofilters are similarly found in aquatic environments or wastewater treatment systems and soil or waste/drinking water treatment systems, respectively (34). As for clade A.3, these *Nitrospira* species are associated more commonly with both natural and agricultural soil environments (34), making their rarity in freshwater biofilters expected based on previously observed niche preferences. Although about half of the detected AmoA sequences are previously known, including cultivated representatives *Ca*. N. nitrosa (AmoA-30) and *Ca*. N. nitrificans (AmoA-68), the remainder represent unknown strains of comammox *Nitrospira*. This suggests much-unexplored diversity in this group, requiring further research to determine if any unique metabolic features exist within these newly detected comammox *Nitrospira* strains.

Despite the ubiquitous and dominant presence of comammox *Nitrospira* within freshwater aquarium biofilters, there were still seven freshwater aquaria whose ammonia oxidizers were dominated by AOA. There are no clear factors indicating why AOA are more abundant than comammox within certain aquaria. It is possible that the differential dominance of these groups in biofilters is simply the result of stochastic community assembly processes, although there may be other deterministic factors at play that were not examined here.

In saltwater aquarium biofilters, the absence of comammox *Nitrospira* reflects current research that has not identified them in marine environments, including a recent study that observed comammox *Nitrospira* abundance was negatively correlated with salinity (35). However, comammox *Nitrospira* has been detected in the brackish, salt marsh, and estuarine environments (36, 37), indicating niche partitioning driven by a salinity tolerance below concentrations of marine environments. Collecting saltwater aquarium biofilters for this study was challenging as fewer saltwater aquarists exist compared with freshwater aquarists who could provide biofilter samples. However, the acquisition of future data sets with a more balanced sampling of salt and freshwater biofilters would be ideal to support conclusions that the role of autotrophic ammonia oxidation is occupied by AOA and AOB in fully marine environments.

### Niche differentiation of ammonia oxidizers in aquarium biofilters

Previously, pH and ammonia concentration were identified as key factors governing ammonia-oxidizing microorganism abundance in the environment (38). Here, our results support observations from earlier studies that comammox *Nitrospira* and AOA are dominant in relatively low ammonia conditions, with several cultivated representatives having high affinities for ammonia including *N. inopinata, Ca*. N. kreftii, *Nitrosopumilus maritimus,* and *Ca*. Nitrosotenuis aquarius (39–41). At this time, there are few cultivated representatives of comammox *Nitrospira* where ammonia affinities have been measured experimentally requiring further research to confirm a generalized high ammonia affinity across all *Nitrospira* spp. In contrast, kinetic studies across many genera of AOA have revealed a wider range of ammonia affinities [K_m(app)_] with some *Nitrososphaerales* (Group I.1b) AOA, such as *Nitrosocosmicus,* having lower ammonia affinities comparable with those of AOB spp. (42). We did observe a higher relative abundance of *Nitrosotenuis* spp. compared with *Nitrosocosmicus* spp. in our freshwater samples, which may reflect the lower ammonia niche occupied by *Nitrosotenuis* spp. in the biofilters. Experimental data so far highlight the importance of ammonia concentrations on ammonia oxidizer abundance in biofilters, which should be evaluated under controlled experimental conditions with varied ammonia concentrations, as most aquaria naturally have low levels of ammonia with well-established nitrifying communities in their biofilms.

Differentiation in microbial community composition at a functional level between saltwater and freshwater was evident among nitrifiers, not only with the presence and absence of comammox *Nitrospira* in fresh and saltwater aquaria, respectively, but also in the AOA, AOB, and NOB species present in the biofilter communities. *Nitrosopumilus* was the only AOA genus detected in saltwater biofilters and was originally discovered in marine aquarium gravel (18). As for freshwater aquaria, AOA from the genera *Nitrosotenuis* and *Nitrosocosmicus* were common, including both *Ca*. Nitrosotenuis aquarius and *Ca*. Nitrosocosmicus hydrocola, which were each discovered in a freshwater aquarium biofilter and a tertiary wastewater treatment system, respectively (41, 43). This similar niche distinction of nitrifiers in saltwater and freshwater was previously observed in aquarium surveys (21) and noted in the structure of nitrifying communities in moving bed biofilm reactors (44). Regarding nitrite oxidation, only a few saltwater biofilter samples had detectable NOB genera belonging to either *Nitrospira* or *Nitrospina* spp. at a low relative abundance (<0.5%) within the community and detectable nitrite in the water (Table S2), suggesting that nitrite oxidizers were not well established within the sampled biofilter material.

The observed correlation between ammonia concentration and comammox *Nitrospira amoA* gene distribution suggests ammonia niche preferences among the clade A comammox *Nitrospira* inhabiting the freshwater biofilters. Similarly, in a study of groundwater-fed rapid sand filters, ammonia concentration explained some of the distribution of comammox *Nitrospira* (based on *amoA* gene sequences) (13). Additionally, the existence of high functional diversity of comammox *Nitrospira* within a system, which has been observed in rotating biological contactors biofilm of a WWTP, might be another factor influencing the distribution and coexistence of comammox *Nitrospira* spp. that could be explored with follow-up metagenomic analyses (9).

The presence of AOA and comammox *Nitrospira* identified via genes for ammonia oxidation suggests that they are performing ammonia oxidation in these systems; however, both have the potential for alternative metabolisms, making it important to confirm the ammonia oxidation activity of AOA and comammox with activity-based experiments. Some AOAs have known potential for mixotrophy, whereas most comammox *Nitrospira* can use different forms of nitrogen, such as urea and cyanate (9, 41, 45). With the potential for alternative metabolisms, it is important that we confirm the ammonia oxidation activity of AOA and comammox with activity-based experiments. Additional factors that were not explored in our study that may contribute to niche differentiation and differential abundance between AOA and comammox include dissolved oxygen (DO) concentrations, which have been noted in other studies where comammox *Nitrospira* are found at high abundance in environments with low DO concentrations (28, 31) and inhibition of ammonia oxidizers by organic compounds and metals (46–48), which should be evaluated in future work.

### Factors influencing aquarium biofilter microbial community composition

In this study, the correlations of freshwater biofilter microbial community composition with temperature, aquarium size, and general water hardness are consistent with previous studies that demonstrated the impact of environmental, biological, and physical factors, such as temperature, filter support material, and fish species in the differentiation of biofilter microbial communities of RAS, water treatment, and aquaponic systems (49–51). The presence of phyla *Proteobacteria*, *Bacteroidota*, and *Planctomycetota* found across all biofilters in this survey in similar proportions was also observed in previous research of freshwater aquaria (52), and freshwater and saltwater RAS biofilters (50, 53, 54). This might suggest a common general microbial community composition found within biofilter systems.

Alongside nitrification, other biofilter microbial community members play significant roles in waste removal from the water to maintain a healthy environment for an aquarium’s animal residents. These roles can include the degradation of organic wastes (e.g., dissolved organic matter) by heterotrophic bacteria and microbially mediated processes like denitrification, anaerobic ammonium oxidation (anammox), methanogenesis, and dissimilatory nitrate reduction to ammonia (DNRA) (53). Denitrification was not measured in our study, but microorganisms capable of denitrification may have been present in the biofilters. Within three freshwater samples (FW-05, FW-37, and FW-47), very low relative abundances (<0.2%) of *Ca*. *Anammoximicrobium* was detected, suggesting the potential for low levels of anammox activity within the freshwater biofilters (55). Although considering the high level of aeration designed to occur in many aquarium biofilters, it is unlikely that such anaerobic metabolisms are commonplace. Overall, the occurrence of these microbiological processes is dependent on the conditions within the biofilter (e.g., dissolved oxygen and pH) and the microbial community composition, which highlights the importance of increasing our understanding of the ecology of aquarium biofilters and factors affecting community assemblage to optimize the functionality of biofilter microbial communities, both in home aquaria and commercial systems.

### Interactions between nitrifiers and heterotrophic biofilter community members

Beyond their primary metabolic functions within the biofilters, relationships between microbial community members could be important to establish and support nitrification activity. Network analysis revealed that many positive connections exist between nitrifiers and other putative heterotrophs within the freshwater aquarium biofilters (Fig. 6), suggesting that the coexistence of these microorganisms may be mutually beneficial. Several *Nitrospira* ASVs have connections with members from the phylum *Myxococcota* (e.g., *Haliangium* spp.), which have been identified as predatory bacteria including in environments like activated sludge systems and freshwater lakes (56–58). We hypothesize that micropredators could benefit nitrifiers, as the predation within the biofilter might lead to reduced competitive pressure for resources (59). Additionally, many heterotrophic species across freshwater biofilters had strong positive connections with various *Nitrospira* and AOA ASVs, leading to the hypothesis that there may be either commensal or mutualistic relationships between these heterotrophs and nitrifiers. Heterotrophs exhibiting growth-promoting effects on AOA and NOB *Nitrospira,* and conversely, growth-promoting effects of nitrifiers on heterotrophs have been identified in previous studies (60–62), supporting the possibility of symbiotic relationships between microbial community members. Mutualistic relationships have also been observed in anammox granules, bacteria associated with the phylum *Chlorobi* appeared to be active protein degraders while also recycling nitrate to nitrite, which are likely beneficial for the activity of their anammox bacteria neighbors (63).

A major challenge in the study of nitrifiers and especially for comammox *Nitrospira* is obtaining pure cultures, as nitrifiers frequently grow in co-cultures alongside other heterotrophic microorganisms, which further supports the hypothesis for cooperative community interactions between nitrifiers and heterotrophs (5, 39). Network analyses suggest the existence of many interactions between biofilter microbial community members; however, these results alone are not sufficient to make strong conclusions regarding microbial interactions, but instead as a hypothesis generation tool to test species interactions that can be tested in future experiments. Overall, more research is needed to elucidate nitrifier-heterotroph interactions between community members and whether their coexistence in biofilms also plays a significant role in the ammonia or nitrite oxidizers that dominate within aquatic biofilter environments.

### Conclusions and future work

This study has further clarified our understanding of ammonia oxidation in home aquarium biofilters, revealing that comammox *Nitrospira* is ubiquitous and with high strain level diversity in freshwater biofilters, where the dominant ammonia oxidizers are either comammox *Nitrospira* or AOA. Future research should further address factors involved in niche differentiation of nitrifiers, elucidating interactions with other microbial community members and exploring group-specific contributions to ammonia oxidation rates in the biofilters. Additionally, we still need to evaluate how ammonia oxidizer distribution may vary between different biofilter substrates and surfaces, as our study focused on either sponge or filter floss material. An improved understanding of this microbially mediated process in aquaria is important to help improve and optimize our current water treatment in both the home aquarium and aquaculture industries and could lead to future development of aquarium biofilter supplements containing comammox *Nitrospira,* improving upon those currently available that contain mainly AOB and NOB, and rarely AOA (21, 25).

## MATERIALS AND METHODS

### Sample collection

Aquarium filter and water samples were collected in 2019 from members of the Kitchener Waterloo Aquarium Society (KWAS), community members, and local pet stores; a total of 38 freshwater and eight saltwater tanks were sampled (Tables S1 and S2). Small pieces of sponge, foam, or floss (as appropriate for each biofilter) were collected using sterile scissors and forceps and then stored in Ziplock bags. During transport to the University of Waterloo, samples were kept on ice and subsequently stored at −70°C prior to DNA extraction. Aquarium water samples were collected alongside filter samples and kept on ice during transport. Prior to storage at −20°C, water samples were distributed into two 50 mL Falcon tubes, one of which was filtered through a 0.45 mm syringe filter for chemical analysis. Participants also provided aquarium information including temperature, maintenance history, water source, and the number and species of fish and plants (Tables S1 and S2).

### DNA extraction

DNA was extracted from aquarium biofilter samples using the DNeasy PowerSoil Kit (Qiagen). Filter samples were thawed, and a ~2 cm^3^ portion from the inside of the sample that had no contact with the collection bag was cut into small pieces and placed into the 2 mL bead-beating tube using sterile scissors and forceps. The extraction was performed following the manufacturer’s protocol, with the addition of a 10 min pre-incubation at 70°C prior to bead beating to help maximize cell lysis. Subsequent homogenization was performed using a FastPrep-24 bead beater (MP Biomedical, Santa Ana, CA) for 45 s at 5.5 m s^−1^. Extracted DNA was visualized on a 1% agarose gel using Gel Red Nucleic Acid Gel Stain (Sigma) and quantified using Qubit dsDNA HS Assay Kit (ThermoFisher Scientific). Extracted DNA samples were stored at −20°C until further use.

### Quantitative PCR

Quantitative PCR (qPCR) was performed on biofilter DNA extracts to determine both 16S rRNA and *amoA* gene copies. The 16S rRNA genes associated with bacteria were quantified using the primer set 341F/518R (64), and Thaumarchaeota/Thermoproteota 16S rRNA genes were amplified with primers 771F/957R (65). The *amoA* genes were amplified using comaA-244F(a-f) and comaA-659R(a-f) to target clade A comammox *Nitrospira* (12), crenamoA-23F/crenamoA-616R to target AOA (66), and amoA1F/amoA2R to target AOB (67). All qPCR amplifications were run as technical duplicates using the CFX96 Real-Time PCR Detection System (Bio-Rad, Hercules, CA, USA). The 10 mL reaction volumes contained 1× SsoAdvanced Universal SYBR Green Supermix (Bio-Rad, Hercules, CA, USA), 5 mg of bovine serum albumin, primers, and 1–10 ng of template DNA. The amount of forward and reverse primer used per sample for amplification varied depending on the primer set. For the crenamoA-23F/crenamoA616R and amoA1F/amoA2R primer sets, 4 pmoles of each primer were used, the 341F/518R primer set used 3 pmoles of each primer, 2 pmoles of each primer was used for the 771F/957R primer set, and 5 pmoles were used for the comaA-244F(a-f) and comaA-659R(a-f) primer pair.

The PCR conditions for the 341F/518R and 771F/957R primer sets involved an initial 3 min denaturation at 98°C, then 35 cycles at 98°C for 30 s followed by a combined annealing and extension step at 55°C for 45 s. For clade A comammox *Nitrospira amoA* gene amplification, an initial denaturation for 3 min at 98°C was also used, followed by 35 cycles at 98°C, 45 sec at 52°C, and 1 min at 72°C. Both the AOA and AOB *amoA* gene amplification began with an initial denaturation step for 3 min at 98°C, followed by 35 cycles of 30 sec at 98°C, 30 sec at 55°C or 60°C for the AOA and AOB amplification, respectively, and 1 min at 72°C. Following all qPCR amplifications, a melt curve was run from 65°C–95°C with 0.5°C interval increases, each lasting for 2 sec. Gel-purified PCR amplicons generated from *Ca*. Nitrosotenuis aquarius, *Nitrosomonas europaea*, and pooled aquarium biofilter DNA templates were used as qPCR standards for AOA, AOB, and comammox *Nitrospira amoA* gene targets, respectively, whereas purified PCR amplicons generated from *Thermus thermophilus* and *Ca*. N. aquarius DNA was used as standards for bacterial and *Nitrososphaeria* 16S rRNA gene targets, respectively (Tables S3 to S4). Starting DNA copy numbers in standards used for qPCR were determined based on the DNA concentrations of the PCR amplicons, with standard curves ranging from 10^0^ to 10^7^ gene copies. Analysis of qPCR data, including quantification and melt curve analysis, was done through CFX Manager Software (version 1.5; Bio-Rad). Efficiencies across all qPCR assays ranged from 82.6%–99.9%, with *R*^2^ values >0.99 for all standard curves. Final qPCR products were also verified using 1% agarose gels. Clade B comammox *Nitrospira amoA* primers (12) were not used for qPCR because no clade B *amoA* gene signal was detected during initial end-point PCR screening using all previously mentioned primer sets (data not shown). Statistical analysis of qPCR gene copy data was done in R (version 4.3.2) to perform non-parametric Wilcoxon rank sum and Kruskal Wallis rank sum tests to test for significant differences between *amoA* gene abundances of all three groups of ammonia oxidizers. Additional post-hoc analysis was done using Dunn’s test function from the FSA (0.9.5) R package. All uncertainty values for qPCR results represent calculated standard deviation (SD) values.

### Water chemistry

Water that was filtered through a 0.45 mm syringe filter prior to storage at −20°C was used for subsequent assays. Total ammonia was quantified using the orthophthaldialdehyde fluorometric assay (68, 69), and the Griess reagent was used to determine nitrite/nitrate concentrations (70). The pH of water samples was measured using a LAQUAtwin pH-33 meter (Horiba Advanced Techno Co., Ltd.). Additionally, general water hardness (GH) and carbonate hardness (KH) were measured using the GH and KH Test Kit (Freshwater; API). General hardness, which is a measure of primarily dissolved magnesium and calcium ions, was quantified using degrees of GH (dGH) where 1 dGH = 17.9 ppm of calcium carbonate equivalents. Carbonate hardness was measured using degrees of KH (dKH) where 1 dKH = 17.9 ppm of CaCO_3_, which measures specifically concentrations of carbonate in the water. Alkalinity was also measured using the MultiTest Marine pH & Alkalinity kit (Seachem). A measure of total alkalinity quantifies the ability of water to buffer or neutralize acid and is expressed here as meq/L, where 1 meq/L = 50 ppm of CaCO_3_.

### 16S rRNA gene sequencing and analysis

Primers 515F-Y and 926R were used to target the V4-V5 region of 16S rRNA genes (71, 72). For each sample, 25 mL PCR amplifications contained 1× ThermoPol buffer, 15 mg of bovine serum albumin, 200 mM of dNTPs, 0.2 mM of both forward and reverse primers, 0.625 units of Hot Start *Taq* DNA Polymerase (New England Biolabs, MA, USA), and 1–10 ng of template DNA. The PCR cycling conditions consisted of an initial denaturation at 95°C for 3 min, followed by 35 cycles of denaturation at 95°C for 30 s, annealing at 50°C for 30 s, and extension at 68°C for 1 min, with a final extension time of 7 min at 68°C. Individual samples were amplified in triplicate, with unique barcoded adapters attached to primers to allow sequencing of pooled samples (73). Negative controls containing no DNA template (NTC) and positive controls (1:1 *Aliivibrio fischeri* and *Thermus thermophilus* DNA) were included as samples during PCR amplification and for sequencing. The resultant triplicate PCR products for each sample were pooled and quantified using a 1% agarose gel stained with GelRed (Biotium, CA, USA). Quantified pooled triplicates were then combined in equimolar concentrations based on gel quantification to create an amplicon library, which was sequenced using the MiSeq Reagent Kit v2 (2 × 250 cycles, Illumina, Canada) on a MiSeq System (Illumina, CA, USA) at the University of Waterloo following manufacturer’s protocols and guidelines. Following demultiplexing of paired-end sequence reads using MiSeq Reporter software version 2.5.0.5 (Illumina), analysis was done using QIIME2 (version 2020.6) implemented through the AXIOME3 pipeline (74). Quality trimming, primer sequence removal, denoising, paired-end sequence merging, chimera removal, and final generation of an amplicon sequence variant (ASV) table was done using DADA2 (75) (Supplemental data file 1). Taxonomic classification of ASVs was done through AXIOME3 using the SILVA database release 138 (76). A phylogenetic tree of ASV sequences was generated using FastTree (77). Additional ASV table analysis was performed using the AXIOME3 pipeline, including rarefaction to the lowest sample count (8417) prior to the calculation of beta diversity metrics (weighted UniFrac, Bray-Curtis) and generation of triplot ordinations. Correlations between environmental variables and beta diversity were determined using the envfit() function in the R vegan package (version 2.6–4) to fit environmental variables onto the ordination space. PERMANOVA testing was performed through QIIME2 (version 2020.6) using “beta-group-significance” available through the “diversity” plugin. All variances for relative abundances shown in Results represent calculated standard deviation values.

### Comammox *Nitrospira amoA* gene sequencing

The *amoA* genes of clade A comammox *Nitrospira* were amplified by PCR for each of the 38 freshwater aquarium biofilter samples using the comaA-244F(a-f) and comaA-659R(a-f) forward and reverse primers following previously established protocols (12). The PCR was set up in triplicate using 25 mL reaction volumes, each containing 1× ThermoPol buffer, 5 mg bovine serum albumin, 200 mM forward and reverse dNTPs, 500 mM of each forward and reverse primer, 0.625 units of *Taq* polymerase, and 1–20 ng of template DNA and amplified using 35 cycles. Alongside the freshwater biofilter samples, *amoA* genes of comammox *Nitrospira* from additional aquarium enrichments containing comammox *Nitrospira* (see Supplementary Information) and an additional biofilm sample from a tertiary treatment system of a Guelph, ON municipal WWTP were amplified for sequencing. The WWTP biofilm sample was used as a positive control and contained two known clade A comammox *Nitrospira* sequences. After amplification, the PCR product was confirmed using gel electrophoresis, and triplicate samples were pooled.

Final *amoA* gene PCR products were sent off to the Centre for Analysis of Genome Evolution and Function (CAGEF) at the University of Toronto (Mississauga, ON). Bead purification was used to clean up PCR products prior to library preparation. The NEBNext Ultra II DNA Library Prep Kit for Illumina (New England Biolabs) was used to generate sequencing libraries, with the addition of barcoded Illumina adapters to the end of the amplified *amoA* gene amplicons. Additional size selection was performed prior to sequencing, and fragment size was verified on a bioanalyzer. The prepared amplicon library was then sequenced (2 × 250 bp) on an MiSeq System following the manufacturer’s protocol. Raw DNA sequence reads are available through the NCBI Sequence Read Archive (PRJNA780914). Demultiplexed raw sequence reads were processed using QIIME2 (version 2020.6) implemented through the AXIOME3 pipeline (74). Similar to 16S rRNA gene sequence data processing, quality trimming, primer removal, denoising, paired-end sequence merging, chimera removal, and final generation of an amplicon sequence variant (ASV) table were done using DADA2 (75). From the ASV table, individual ASVs were further sorted using a custom R script to identify and combine reverse complement ASV pairs present in the data. These reverse complement pairs were present because of the non-preferential ligation of barcodes to either end of the PCR products amplified for sequencing. After merging reverse complement sequence pairs, ASV-translated nucleotide sequences were analyzed using tblastn available through NCBI Web BLAST against the AmoA amino acid sequence of *N. inopinata* using default algorithm parameters. Any sequences that did not align with the *N. inopinata* AmoA amino acid sequence were removed from the data, as they were not true comammox *amoA* gene amplicons. Alignments with *amoA* gene sequences from other ammonia oxidizers such as *N. europaea* (AOB) and *N. maritimus* (AOA), which align with some identity to the AmoA sequence of *N. inopinata*, were used as controls to confirm that any sequence that did not align was an off-target product in the amplicon sequence data. The final *amoA* gene ASV table was then used for subsequent data analysis (Supplemental data file 2). The ASVs were collapsed based on 100% amino acid identity, and the resulting unique amino acid sequences were used to generate a phylogenetic tree with MEGA X (version 10.1.8) (78). A heatmap of the collapsed *amoA* amplicon data was generated through R (version 4.1.2) using the ggdendro (0.1.23), ggtree (3.2.1), and ggplot2 (3.4.1) packages. The triplot analysis and visualization of the amplicon data were also done with a custom R script implementing the vegan (2.6–4), ggplot2 (3.4.1), and ecodist (2.0.9) packages. Beta diversity of AmoA sequences across samples was calculated using the ‘vegdist’function from vegan. The ‘envfit’ function from vegan was used to calculate correlations and fit environmental variables (pH, temperature, dGH, dKH, months since sponge replacement, alkalinity, ammonia, nitrite, and nitrate) onto the ordination space. Additionally, the distribution of individual *amoA* gene sequences across samples was fitted to the ordination using the ‘wascores’ function from the vegan package, which computes the weighted averages for a variable across samples in an ordination.

### Network analysis

To explore direct interactions between microbial community members, nitrifiers, and metadata within freshwater biofilters, network analysis was performed using FlashWeave, which predicts ecological interactions between microbial community members while removing spurious correlations that result from indirect network edges (24). To help prevent spurious associations during network analysis, the freshwater ASV table was filtered to remove any ASVs that were not present in at least three of the samples. Additionally, because FlashWeave is unable to handle missing values, samples that were missing large portions of metadata, FW-22, FW-23, and FW-24, were removed from the ASV table prior to filtration. The filtered table was used to generate a correlation network using the FlashWeave–sensitive mode in FlashWeave (version 1.6), with the learn_network function using the parameters, sensitive = true, heterogeneous = false, max_k = 3, alpha = 0.05, and sparse = false. The output network was then filtered using Cytoscape to select only nodes corresponding to genera of known nitrifiers (e.g., “*Nitro”* or “*Nitroso”* containing genera) and the first neighbors of those nodes, along with any edges existing between these selected nodes. These selected nodes and edges comprised the final visualized network. Filtered and original network tables were manually checked to ensure that all ASVs of known nitrifier genera were included. All FlashWeave network analyses were run using Julia (version 1.9.2) (79), and we used Cytoscape (version 3.10.0) for network visualization (80).

## Data Availability

All DNA sequences were deposited in the NCBI Sequence Read Archive under BioProject accession number PRJNA780914.

## SUPPLEMENTAL MATERIAL

Data file S1 aem.00104-24-s0001.txt ASV table (16S rRNA gene).

Data file S2 aem.00104-24-s0002.txt ASV table (*amoA* gene).

Supplemental material aem.00104-24-s0003.docx Supplemental methods, Tables S1 to S7, and Fig. S1 and S2.

ASM does not own the copyrights to Supplemental Material that may be linked to, or accessed through, an article. The authors have granted ASM a non-exclusive, world-wide license to publish the Supplemental Material files. Please contact the corresponding author directly for reuse.
