# Supplementary material for: Comammox Nitrospira among dominant ammonia oxidizers within aquarium biofilter microbial communities
Source: Appl Environ Microbiol. 2024 Jun 20;90(7):e00104-24. doi: 10.1128/aem.00104-24 (PMC11267875; doi:10.1128/aem.00104-24)
Supplement: Supplemental material — Supplemental methods, Tables S1 to S7, and Fig. S1 and S2. [file aem.00104-24-s0003.docx]

**Supplementary Information**

**Comammox *Nitrospira* among dominant ammonia oxidizers within aquarium biofilter microbial communities**

Michelle M. McKnight^1^ and Josh D. Neufeld^1^

^1^Department of Biology, University of Waterloo, Waterloo, Ontario, Canada

**qPCR methods**

*Generation of qPCR gene standards*

DNA templates used to generate standard curves for the comammox *amoA*, AOA *amoA*, AOB *amoA*, and 16S rRNA gene thaumarcheotal qPCR were generated using PCR with the same primers described in the main manuscript. Following PCR amplification of these target sequences, PCR products were purified using the Wizard SV Gel and PCR Clean-Up System (Promega). For the 16S rRNA gene qPCR standard, the target gene was amplified from a pUC57-Kan vector containing the target 16S rRNA gene fragment from *Thermus thermophilus* flanked by M13 primers. The M13 primers were used to amplify the 16S rRNA gene target and following PCR amplification the PCR product was also purified as described for the other four DNA template targets. Standards were stored in aliquots at concentrations of around 10^10^ copies/μL at -20°C. The DNA concentration of standard aliquots were measured on the same day the qPCR was performed using a Qubit dsDNA HS Assay Kit (ThermoFisher Scientific). Measurements were done in duplicate using 4 μL of sample, and the average concentration was used to determine the copy number concentrations for the qPCR standard curves alongside the molecular mass of each standard amplicon sequence as described below.

For the AOA, AOB, bacterial, and archaeal targets, exact sequences of the amplicons were known, and these sequences were used to determine the exact copy numbers added to each qPCR based on the molecular weight of the fragment (Table S1-S2). Since the standard for clade A comammox *Nitrospira* was amplified from a mixture of different comammox *amoA* sequences from aquaria, a molecular weight of an exact sequence could not be used to determine the copy number. Therefore, the average molar mass per bp of 650 g/mol/bp was used to determine the copy number concentrations added to the qPCRs. Specifically, the ThermoFisher Sci DNA copy number and dilution calculator (<https://www.thermofisher.com/ca/en/home/brands/thermo-scientific/molecular-biology/molecular-biology-learning-center/molecular-biology-resource-library/thermo-scientific-web-tools/dna-copy-number-calculator.html>) was used to determine copy number based on the concentration of the standard DNA template solution. For the other standards with known DNA sequences, the Science Primer copy number calculator was used to determine copy number based on the exact molecular weight of the fragment and DNA concentration ([http://www.scienceprimer.com/copy-number-calculator-or-realtime-pcr](http://www.scienceprimer.com/copy-number-calculator-for-realtime-pcr)).

*Preparation of qPCR standard curve dilution series and detection limits*

The standard curve DNA samples were prepared using a freshly thawed standard aliquot that had a concentration of ~10^10^ copies/μL. Standard dilutions were prepared in a 10-old dilution series from magnitudes of 10^9^ copies/μL to 10^0^ copies/μL in 100 μL volumes. Each dilution was made with 10 mM Tris-HCl buffer containing 0.05% Tween-20 to minimize adherence of any DNA to the tube.

Detection limits for each qPCR were defined as the lowest magnitude standard that was present in the final standard curve for each gene target. Although we did include standards at a magnitude of 10^0^ copies, none of these amplified in a successful manner and were omitted from the standard curve range. The detection limits for each of the five assays were as follows: for the *amoA* gene targets the detection limits for AOA, AOB, and comammox *Nitrospira* were 50, 70, and 75 copies per reaction respectively. For the 16S rRNA archaeal gene target, the detection limit was 10 copies. While the lowest amplified standard for the bacterial 16S rRNA gene target was ~40 copies, this fell within the range of copies detected in the NTC (as background contamination) of 30-60 copies. Therefore, we set the detection limit for this gene target at the next lowest standard of 400 copies, which was still well below the average range of detection at a magnitude of 10^5^ copies.

*Testing for inhibition of qPCR*

To test for PCR inhibitors in extracted DNA samples used as template for qPCR, we conducted an inhibition assay based on a previously described protocol (1) with modifications. We used an in-house 16S rRNA gene standard template containing P5-P7 Illumina adapter sequences that are used during sequencing library preparation to amplify a 164 bp target fragment (Table S4). The qPCRs were run in technical duplicates for samples, with each reaction containing 1X SsoAdvanced Universal SYBR Green Supermix (Bio-Rad, Hercules, CA, USA), 5 μg of bovine serum albumin, primers, 1-10 ng of template DNA, and 5 pmol of both the P5 forward primer and P7 reverse primer to a final volume of 15 μL. Each sample containing template DNA was also spiked with 10,000 copies of the P5-P7 16S rRNA standard control sequence target. Additionally, eight replicate reactions were run as “control samples” containing only ~10,000 copies of the standard to provide a baseline for where the Cq value is expected to be for this target copy number. The conditions for qPCR were as follows: initial 3 min denaturation at 98°C, then 40 cycles at 98°C for 10 s followed by a combined annealing and extension step at 60°C for 30 s, with a final melt curve was run from 65-95°C with 0.5°C interval increases, each lasting for 2 sec. The qPCR was run using a CFX96 Real-Time PCR Detection System (Bio-Rad, Hercules, CA, USA) and analyzed using CFX Maestro Software (version 2.3; Bio-Rad).

Control samples containing 10,000 copies of the standard template had an average Cq value of 22.3 ± 0.2 (min 22.0, max 22.6; n = 16). Standard template spiked with aquarium DNA had an average Cq value of 22.7 ± 0.3 (min 22.1, max 23.5; n = 46). All Cq values for template spiked with aquarium DNA were within 1 cycle of the non-spiked controls, demonstrating minimal PCR inhibition by co-extracted inhibitors.

**Enrichment culture methods**

The comammox *Nitrospira* aquarium biofilter enrichment cultures included in the *amoA* (Aq-C1 and Aq-C2) originate from biomass from a freshwater aquarium biofilter. The original parent culture was started by taking 5 mL of water directly from within the biofilter while it was not running and inoculating it into 45 mL of AOM medium containing 0.5 mM NH_4_Cl in a 100 mL Schott bottle (2). The enrichment was incubated in the dark at room temperature. After ~5 weeks, the culture had oxidized all the ammonia to both nitrite and nitrate and was re-fed ammonia at the same concentration of 0.5 mM as was originally present in the AOM medium. Two weeks later, DNA extracted from the culture was used to run PCR with the comaA-P primers targeting the comammox *Nitrospira* clade A *amoA* gene (3). The PCR detected the presence of comammox *Nitrospira amoA* genes, indicating their presence in the enrichment culture.

Given the detection of comammox *Nitrospira* and active ammonia oxidation, we decided to create a sub-culture from this original enrichment culture by transferring 2 mL of the parent culture into 48 mL of fresh AOM medium (0.5 mM NH_4_Cl), and an additional 5 mL of water from the original aquarium filtered through a 0.1 μm syringe filter in a new 100 mL Schott bottle. Incubation of the cultures was continued at room temperature in the dark. After 2 weeks, the new sub-culture had oxidized all ammonia into nitrate and was sub-cultured again following the same protocol as just described. This newest sub-culture was our Aq-C1 enrichment culture. The Aq-C1 enrichment culture was continually re-fed 25 μL of 1M NH_4_Cl every few weeks once it had oxidized all the ammonia into nitrate and PCR did detect the presence of comammox *Nitrospira*. Around 2 months after incubation, Aq-C1 was sub-cultured again following the same protocol – this newest subculture was Aq-C2. After several weeks, Aq-C2 had detectable comammox *Nitrospira* (via PCR) and was oxidizing all provided ammonia to nitrate, indicative of comammox activity.

Both Aq-C1 and Aq-C2 enrichment cultures were re-fed 25 μL of 1M NH_4_Cl every few weeks as they oxidized the ammonia to nitrate. They were continually kept in the dark at room temperature. When enrichment cultures had a nitrate concentration greater than 500 mg/L N-NH_3_, then most of the AOM medium was removed from the Schott bottle, leaving behind the solid calcium carbonate, and replaced with fresh AOM medium to decrease the overall nitrate concentration. Approximately 6 months after starting Aq-C2, DNA was extracted from both Aq-C1 and Aq-C2, which were both still actively oxidizing ammonia and had detectable comammox *Nitrospira.* This extracted enrichment culture DNA was used for the comammox *Nitrospira* *amoA* sequencing described in the main manuscript. All DNA extractions were performed on 2 mL of enrichment cultures using the DNeasy UltraClean Microbial Kit (Qiagen) following the manufacturer’s protocol.

**References**

1. Nolan T, Hands RE, Ogunkolade BW, Bustin SA. 2006. SPUD: a qPCR assay for the detection of inhibitors in nucleic acid preparations. Anal Biochem 351:308-310.
2. Daims H, Lebedeva E V., Pjevac P, Han P, Herbold C, Albertsen M, Jehmlich N, Palatinszky M, Vierheilig J, Bulaev A, Kirkegaard RH, Von Bergen M, Rattei T, Bendinger B, Nielsen PH, Wagner M, Bergen M von, Rattei T, Bendinger B, Nielsen PH, Wagner M. 2015. Complete nitrification by *Nitrospira* bacteria. Nature 528:504–509.
3. Pjevac P, Schauberger C, Poghosyan L, Herbold CW, Kessel MA van, Daebeler A, Steinberger M, Jetten MS, Lücker S, Wagner M, Daims H. 2017. *amoA*-targeted polymerase chain reaction primers for the specific detection and quantification of comammox *Nitrospira* in the environment. Front Microbiol 8:1508.
4. Muyzer G, de Waal EC, Uitterlinden AG. 1993. Profiling of complex microbial populations by denaturing gradient gel electrophoresis analysis of polymerase chain reaction-amplified genes coding for 16S rRNA. Appl Environ Microbiol 59:695–700.
5. Ochsenreiter T, Selezi D, Quaiser A, Bonch-Osmolovskaya L, Schleper C. 2003. Diversity and abundance of Crenarchaeota in terrestrial habitats studied by 16S RNA surveys and real time PCR. Environ Microbiol 5:787–797.
6. Rotthauwe J-H, Witzel K-P, Liesack W. 1997. The ammonia monooxygenase structural gene *amoA* as a functional marker: molecular fine-scale analysis of natural ammonia-oxidizing populations. Appl Environ Microbiol 63:4704–4712.
7. Tourna M, Freitag TE, Nicol GW, Prosser JI. 2008. Growth, activity and temperature responses of ammonia-oxidizing archaea and bacteria in soil microcosms. Environ Microbiol 10:1357–1364.

**Table S5.** Gene copies for both *amoA* and 16S rRNA gene targeted qPCRs for each sample. Copies are expressed per ng of extracted DNA.

| Sample ID | Sampling date | Location | City | Aquarium type | Filter material | Live plants (y/n) | Added supplements (y/n) | Antibiotic treatment  (< 6 mo.) | Water source | Fish type | Aquarium age (years) | Fish | Last sponge replacement (months) | Frequency of water changes (weeks) |
| --- | --- | --- | --- | --- | --- | --- | --- | --- | --- | --- | --- | --- | --- | --- |
| FW-01 | 03/05/2019 | Residential 1 | Waterloo | Fresh | Sponge | Yes | No | No | Tap water | Discus, angel | 7 | 12 | 12 | 1 |
| FW-02 | 03/05/2019 | Residential 2 | Kitchener | Fresh | Sponge | Yes | No | No | Tap water | Guppies | 0.5 | 10 | Never | 1 |
| FW-03 | 03/05/2019 | Residential 3 | Guelph | Fresh | Sponge | No | No | No | Tap water | Cichlids, pleco | 3 | 37 | 12 | 4 |
| FW-04 | 03/05/2019 | Residential 3 | Guelph | Fresh | Sponge | Yes | No | No | Tap water | Mixed tropical | 2 | 67 | 12 | 4 |
| FW-05 | 03/05/2019 | Residential 4 | Drayton | Fresh | Sponge | No | No | No | Tap water | Cichlids, petricola | 9 | 10 | 60 | 1 |
| FW-06 | 03/05/2019 | Residential 4 | Drayton | Fresh | Sponge | No | No | No | Tap water | Cichlid | 9 | 5 | 60 | 1 |
| FW-07 | 03/05/2019 | Residential 5 | Drayton | Fresh | Sponge | No | No | No | Tap water | Guppies, pleco | 0.1 | 8 | 1 | 4 |
| FW-08 | 03/05/2019 | Residential 5 | Waterloo | Fresh | Sponge | Yes | No | No | Tap water | Guppies, pleco | 2 | 20 | Never | 2 |
| FW-09 | 03/05/2019 | Residential 5 | Waterloo | Fresh | Sponge | Yes | No | No | Tap water | Barbs, pleco | 1 | 13 | Never | 4 |
| FW-10 | 03/05/2019 | Residential 6 | Fergus | Fresh | Floss | Yes | No | No | Tap water | Cichlid, pleco | 0.5 | 2 | Never | 8 |
| FW-11 | 03/05/2019 | Residential 6 | Fergus | Fresh | Floss | Yes | Yes | Yes | Tap water | Mixed tropical | 1 | 25 | Never | 4 |
| FW-12 | 03/09/2019 | Residential 7 | Mississauga | Fresh | Floss | No | No | No | Tap water | Cichlid | 0.5 | 1 | 1 | 5 |
| FW-13 | 03/09/2019 | Residential 7 | Mississauga | Fresh | Sponge | No | No | No | Tap water | Turtle | 9 | 1 | 1 | 4 |
| FW-14 | 03/18/2019 | Residential 8 | Waterloo | Fresh | Sponge | No | No | No | Tap water | Cichlid | 12 | 3 | 8 | 4 |
| FW-15 | 04/02/2019 | Residential 9 | Drayton | Fresh | Floss | Yes | No | No | Tap water | Mixed tropical | 3 | 20 | 6 | 4 |
| FW-16 | 04/02/2019 | Residential 5 | Drayton | Fresh | Sponge | Yes | No | No | Tap water | Guppies, plecos | 0.3 | 30 | 4 | 4 |
| FW-17 | 04/02/2019 | Residential 10 | Kitchener | Fresh | Floss | No | No | No | Tap water | Endlers, crayfish | 4 | 20 | 24 | 2 |
| FW-18 | 04/02/2019 | Residential 11 | Cambridge | Fresh | Floss | Yes | Yes | No | Tap water | Plecos | 0.75 | 10 | 1 | 1 |
| FW-19 | 04/02/2019 | Residential 11 | Cambridge | Fresh | Floss | No | Yes | No | Tap water | Cichlids | 0.3 | 10 | 1 | 1 |
| FW-20 | 04/02/2019 | Residential 12 | Kitchener | Fresh | Sponge | Yes | Yes | No | Tap water | Cichlids | 6 | 12 | Never | 2 |
| FW-21 | 04/02/2019 | Residential 14 | Guelph | Fresh | Sponge | Yes | No | No | Tap water | Danios | 0.75 | 4 | Never | 4 |
| FW-22 | 04/02/2019 | Residential 15 | Waterloo | Fresh | Sponge | No | No | No | Tap water | Unknown | 2 | Unknown | Unknown | Unknown |
| FW-23 | 04/02/2019 | Residential 15 | Waterloo | Fresh | Sponge | No | No | No | Tap water | Unknown | 2 | Unknown | Unknown | Unknown |
| FW-24 | 04/02/2019 | Residential 15 | Waterloo | Fresh | Sponge | No | No | No | Tap water | Unknown | 2 | Unknown | Unknown | Unknown |
| FW-25 | 04/02/2019 | Residential 16 | Guelph | Fresh | Sponge | Yes | No | No | Tap water | Mixed tropical | 0.5 | 75 | Never | 1 |
| FW-26 | 04/02/2019 | Residential 16 | Guelph | Fresh | Sponge | Yes | No | No | Tap water | Cichlids | Unknown | 5 | 2 | 1 |
| FW-27 | 04/02/2019 | Residential 16 | Guelph | Fresh | Sponge | No | No | No | Tap water | Cichlids | 0.5 | 30 | Never | 1 |
| FW-28 | 04/02/2019 | Residential 16 | Guelph | Fresh | Sponge | No | No | No | Tap water | Mixed tropical | 1 | 25 | Never | 1 |
| FW-29 | 04/02/2019 | Residential 3 | Guelph | Fresh | Sponge | Yes | No | No | Tap water | Cichlids | 1 | 103 | 9 | 4 |
| FW-30 | 04/02/2019 | Residential 3 | Guelph | Fresh | Floss | No | No | No | Tap water | Cichlids, plecos | 1 | 40 | 5 | 4 |
| FW-31 | 04/02/2019 | Residential 17 | Waterloo | Fresh | Sponge | Yes | Yes | No | Ground and RO water | Mixed tropical | 3 | 50 | Never | 4 |
| FW-32 | 04/02/2019 | Residential 18 | Waterloo | Fresh | Sponge | No | No | No | Tap water | Guppies, tetras | 2 | 12 | Never | 2 |
| FW-33 | 04/02/2019 | Residential 18 | Waterloo | Fresh | Sponge | Yes | No | No | Bottled water | *Microctenopoma* | 0.5 | 2 | Never | 3 |
| FW-34 | 04/02/2019 | Residential 18 | Waterloo | Fresh | Sponge | No | No | No | Bottled water | Mixed tropical | 1 | 30 | Never | 3 |
| FW-35 | 04/02/2019 | Residential 18 | Waterloo | Fresh | Sponge | Yes | No | No | Tap and distilled water | *Cynodorichthys* | 0.5 | 2 | Never | 3 |
| FW-36 | 05/02/2019 | Residential 19 | Kitchener | Fresh | Sponge | Yes | No | No | RO water | Tetras, raboras | 13 | 8 | 6 | 3 |
| FW-37 | 05/12/2019 | Residential 7 | Mississauga | Fresh | Sponge | No | No | No | Tap water | Turtle | 9 | 1 | 1 | 4 |
| FW-38 | 06/05/2019 | Residential 22 | Unknown | Fresh | Sponge | Unknown | Unknown | Unknown | Unknown | Unknown | Unknown | Unknown | Unknown | Unknown |
| SW-01 | 04/02/2019 | Residential 13 | Guelph | Salt | Floss | Yes | No | No | Tap water | Damsels, clownfish | 3 | 8 | 4 | 4 |
| SW-02 | 05/13/2019 | Residential 20 | Kitchener | Salt | Sponge | Yes | No | No | Tap water | Mixed marine | 13 | 12 | Never | 8 |
| SW-03 | 05/17/2019 | Retail 1 | Kitchener | Salt | Floss | Yes | No | No | RO water | Mixed marine | 1.5 | 10 | 0.25 | 1 |
| SW-04 | 05/17/2019 | Retail 1 | Kitchener | Salt | Floss | Yes | No | No | Tap water | Mixed marine | 1.5 | 12 | 0.25 | 1 |
| SW-05 | 05/17/2019 | Retail 1 | Kitchener | Salt | Floss | No | No | Yes | Tap water | Tangs, wrasse | 1.5 | 3 | 0.25 | 1 |
| SW-06 | 05/17/2019 | Retail 1 | Kitchener | Salt | Floss | No | Yes | Yes | Tap water | Mixed marine | 0.5 | 150 | 0.25 | 1 |
| SW-07 | 05/17/2019 | Retail 1 | Kitchener | Salt | Floss | Unknown | Unknown | Unknown | Unknown | None | Unknown | Unknown | Never | 1 |
| SW-08 | 05/21/2019 | Residential 21 | Unknown | Salt | Floss | Unknown | Unknown | Unknown | Unknown | Unknown | Unknown | Unknown | Unknown | Unknown |

*Antibiotic dosage was not provided by participants.

**Table S1.** Summary of aquarium sample metadata, including information on aquarium type, maintenance, location, number of fish, and sampling dates.

**Table S2.** Water chemistry, temperature, and aquarium size data for all aquarium biofilter samples collected.

| ID | Size (gallons) | Temperature (ºC) | pH | Alkalinity (meq/L) | dGH | dKH | Total NH_3_-N (μg/L) | NO_2_^-^-N (μg/L) | NO_3_^-^-N (μg/L) |
| --- | --- | --- | --- | --- | --- | --- | --- | --- | --- |
| FW-01 | 120 | 28.0 | 8.6 | 14.0 | 3 | 20 | 16.7 | 21.6 | 2394.0 |
| FW-02 | 15 | 25.0 | 8.1 | 14.0 | 21 | 10 | 0.0 | 0.0 | 4274.5 |
| FW-03 | 55 | 22.0 | 8.0 | 4.5 | 30 | 7 | 0.0 | 19.9 | 27829.2 |
| FW-04 | 29 | 24.0 | 8.0 | 2.5 | 31 | 7 | 12.5 | 45.1 | 39762.7 |
| FW-05 | 30 | 24.4 | 8.3 | 3.5 | 32 | 9 | 0.0 | 0.0 | 4127.9 |
| FW-06 | 20 | 24.4 | 8.2 | 3.0 | 32 | 7 | 0.0 | 14.1 | 5097.5 |
| FW-07 | 10 | 25.6 | 8.2 | 4.0 | 14 | 11 | 16.7 | 2013.6 | 2262.0 |
| FW-08 | 20 | 24.4 | 8.1 | 4.5 | 16 | 13 | 3.7 | 13.2 | 6225.1 |
| FW-09 | 30 | 24.4 | 8.2 | 4.0 | 15 | 11 | 20.3 | 0.0 | 7403.5 |
| FW-10 | 29 | 25.6 | 8.5 | 7.5 | 3 | 18 | 0.0 | 0.0 | 30337.0 |
| FW-11 | 75 | 25.0 | 8.4 | 5.0 | 2 | 13 | 0.3 | 0.0 | 17516.9 |
| FW-12 | 90 | 23.9 | 6.8 | 0.0 | 27 | 1 | 1.8 | 16.3 | 21473.1 |
| FW-13 | 5 | 20.0 | 8.4 | 5.0 | 31 | 15 | 232.5 | 94.7 | 4425.8 |
| FW-14 | 110 | 26.7 | 8.3 | 3.0 | 24 | 11 | 0.0 | 0.0 | 4313.0 |
| FW-15 | 50 | 23.9 | 8.7 | 7.5 | 25 | 20 | 25.8 | 0.0 | 9535.9 |
| FW-16 | 50 | 25.6 | 8.6 | 4.5 | 18 | 12 | 0.0 | 20.3 | 8203.7 |
| FW-17 | 5 | 20.0 | 8.1 | 1.5 | 25 | 10 | 12.0 | 0.0 | 5149.2 |
| FW-18 | 65 | 26.0 | 8.1 | 2.5 | 31 | 10 | 0.0 | 18.0 | 3364.0 |
| FW-19 | 90 | 26.0 | 8.3 | 4.0 | 33 | 12 | 0.7 | 15.7 | 8223.9 |
| FW-20 | 110 | 25.6 | 8.1 | 2.5 | 28 | 9 | 22.9 | 0.0 | 2756.5 |
| FW-21 | 5 | 25.0 | 9.3 | 3.5 | 17 | 7 | 0.0 | 0.0 | 15.3 |
| FW-22 | unknown | 27.7 | 7.5 | 2.0 | 52 | 4 | 250.8 | 233.0 | 106676.6 |
| FW-23 | unknown | 27.7 | 8.3 | 7.5 | 46 | 17 | 109.0 | 246.2 | 37921.3 |
| FW-24 | unknown | 27.7 | 8.1 | 4.0 | 38 | 8 | 70.6 | 46.8 | 46290.2 |
| FW-25 | 125 | 27.0 | 8.6 | 12.0 | 4 | 28 | 59.7 | 15.0 | 14986.4 |
| FW-26 | 15 | 22.0 | 8.6 | 10.5 | 3 | 23 | 14.9 | 0.0 | 1004.8 |
| FW-27 | 20 | 28.8 | 8.6 | 16.5 | 5 | 38 | 16.6 | 0.0 | 90721.8 |
| FW-28 | 155 | 27.3 | 8.1 | 4.5 | 8 | 13 | 54.2 | 0.0 | 34680.7 |
| FW-29 | 29 | 26.7 | 8.2 | 4.0 | 39 | 12 | 0.0 | 0.0 | 1976.2 |
| FW-30 | 75 | 23.9 | 8.3 | 4.0 | 41 | 9 | 0.0 | 17.1 | 27163.0 |
| FW-31 | 46 | 23.9 | 7.9 | 1.5 | 19 | 3 | 0.0 | 0.0 | 6683.2 |
| FW-32 | 10 | 19.3 | 8.0 | 4.0 | 17 | 10 | 0.0 | 0.0 | 899.5 |
| FW-33 | 10 | 19.3 | 8.2 | 0.5 | 2 | 1 | 0.0 | 0.0 | 62.9 |
| FW-34 | 10 | 19.5 | 6.2 | 0.5 | 4 | 1 | 650.6 | 0.0 | 4599.3 |
| FW-35 | 10 | 19.7 | 6.9 | 0.5 | 1 | 2 | 0.0 | 0.0 | 849.5 |
| FW-36 | 10 | 25.0 | 7.1 | 0.5 | 40 | 3 | 0.0 | 0.0 | 1012.2 |
| FW-37 | 5 | 20.0 | 8.6 | 7.0 | 46 | 18 | 237.8 | 40.1 | 4943.2 |
| FW-38 | unknown | unknown | 6.5 | 1.5 | 36 | 4 | 136.9 | 1029.4 | 60340.8 |
| SW-01 | 55 | 22.6 | 8.0 | 4.0 | n/a | 11 | 16.2 | 0.0 | 24861.7 |
| SW-02 | 180 | 25.6 | 8.0 | 5.0 | n/a | 14 | 0.0 | 42.4 | 59595.8 |
| SW-03 | 240 | 25.6 | 8.1 | 4.5 | n/a | 12 | 0.0 | 42.4 | 2365.4 |
| SW-04 | 280 | 25.6 | 8.1 | 8.0 | n/a | 22 | 299.8 | 221.5 | 9156.8 |
| SW-05 | 170 | 25.6 | 8.3 | 6.5 | n/a | 16 | 8.2 | 0.0 | 21177.8 |
| SW-06 | 200 | 25.6 | 8.2 | 4.5 | n/a | 13 | 45.4 | 14.0 | 4082.3 |
| SW-07 | unknown | 25.6 | 8.1 | 5.5 | n/a | 15 | 12.7 | 45.2 | 3412.8 |
| SW-08 | unknown | unknown | 7.7 | 12.5 | n/a | 36 | 362.4 | 8231.9 | 1564.5 |

**Table S3.** qPCR standard DNA template information for all five gene targets

| **Target gene** | **Primers** | **Primer reference** | **Standard source** | **Amplicon size (bp)** | **Fragment mass (g/mol)*** |
| --- | --- | --- | --- | --- | --- |
| 16S rRNA bacterial | 341F/518R | Muyzer 1993 | *Thermus thermophilus* | 719 | 444231 |
| 16S rRNA archaeal | 771F/957R | Ochsenreiter et al. 2003 | *Ca.* Nitrosotenuis aquarius | 227 | 140151 |
| AOB *amoA* | amoA1F/amoA2R | Rotthuwe et al. 1997 | *Nitrosomonas europaea* | 491 | 303255 |
| AOA *amoA* | crenamoA23F/  616R | Tourna et al. 2008 | *Ca.* Nitrosotenuis aquarius | 629 | 388505 |
| CMX *amoA* | comaAF/comaAR pooled | Pjevac et al. 2017 | Aquarium samples (4 amplicons pooled for template) | 415 | **269750 |

* Fragment molecular masses correspond to the sequences listed in Table S2 below.

** Calculated based on average molar mass of 650 g/mol/bp as sequence of DNA amplified was variable

**Table S4.** qPCR standard gene sequences with location of target forward and reverse primers indicated in bold. Amplified sequence consists or region between and inclusive of bolded primer sequences.

| Standard source | Sequence |
| --- | --- |
| *Thermus thermophilus*  341F/518R gene standard | GTAAAACGACGGCCAGTGAATTCGAGCTCGGTACCTCGCGAATGCATCTAGATATCGGATCCCGGGCCCGTCGACTGCAGAGGCCTGCATGCAACGGGCCCCACT**CCTACGGGAGGCAGCAG**TTAGGAATCTTCCGCAATGGGCGCAAGCCTGACGGAGCGACGCCGCTTGGAGGAAGAAGCCCTTCGGGGTGTAAACTCCTGAACCCGGGACGAAACCCCCGACGAGGGGACTGACGGTACCGGGGTAATAGCGCCGGCCAACTCCGTG**CCAGCAGCCGCGGTAAT**ACGGAGGGCGCGAGCGTTACCCGGATTCACTGGGCGTAAAGGGCGTGTAGGCGGCCTGGGGCGTCCCATGTGAAAGACCACGGCTCAACCGTGGGGGAGCGTGGGATACGCTCAGGCTAGACGGTGGGAGAGGGTGGTGGAATTCCCGGAGTAGCGGTGAAATGCGCAGATACCGGGAGGAACGCCGATGGCGAAGGCAGCCACCTGGTCCACCCGTGACGCTGAGGCGCGAAAGCGTGGGGAGCAAACCGGATTAGATACCCGGGTAGTCCACGCCCTAAACGATGCGCGCTAGGTCTCTGGGTCTCCTGGGGGCCGAAGCTAACGCGTTAAGCGCGCCGCCTGGGGAGTACGGCCGCAAGGCTGAAACTCAAAGGAATTGACGGGGGCCCGCACAAAGCTTGGCGTAATCATGGTCATAGCTGTTTCCTG |
| *Ca.* Nitrosotenuis aquarius  771F/957R gene standard | **ACGGTGAGGGATGAAAGCT**GGGGGAGCAAACCGGATTAGATACCCGGGTAGTCCCAGCTGTAAACGATGCAGACTCGGTGATGCATTGGCTTGTGGCCAATGCAGTGCCGCAGGGAAGCCGTTAAGTCTGCCGCCTGGGAAGTACGTACGCAAGTATGAAACTTAAAGGAATTGGCGGGGGAGCACCACAAGGGGTGAAGCCTGCGGTT**CAATTGGAGTCAACGCCG** |
| *Nitrosomonas europaea*  amoA1F/amoA2R gene standard | **CCCCTCTGGAAAGCCTTCTTC**ACCGAATGCGGTAACATCATTGCGATGTACGATACGACCTCTTTTACCTTTAACGTAGAAAAAGGCTGTACAGTAAACTTTTCCAAGATACCACCATACGGTGAACATCAACATTGATACGAACGCAGAGAAGAATGCTGCAATAACTGTGGTATGACCACCAAAGGTACGCAGTGAACCTTGCTCAATATGACGAACATACTCGGGTGTACCTGTACGAACATACAGATGTCCCATGTAATCAGCCATCGACAGCAATGTGCCTTCTACAACGATTGGCAAATGGGTTGGTCCAAAAATCGGCCAGTTACCCGGATAGAACAGCAGACCGAAGAATCCACCTCCAACCAGAGCCGTCACCAGCCAGTTGCGTGTCAGATACAGCGTGAAGTCCAGCATCAGCGCACCCGGAAGCATAATGCCCGGTGTTACGAAGTTGATGGGGTAGTGTG**ACCACCAGTAGAATCCCC** |
| *Ca.* Nitrosotenuis aquarius  crenamoA23F/616R | **ATGGTCTGGCTTAGACG**ATGTACGCACTACTTGTTCATAGTAGTCGTAGCAGTCAACTCAACCCTGCTTACAATCAACGCAGGAGACTACATCTTCTACACTGACTGGGCATGGACTTCGTATGTCGTGTTCTCAATATCACAGACATTGATGTTGGTGGTAGGTGCAACTTACTATCTGACATTTACCGGAGTTCCAGGAACCGCAACATACTACGCGCTGATTATGACCGTGTATACATGGATCGCAAAAGGCGCATGGTTTGCTCTAGGTTACCCATATGACTTCATTGTTACACCAGTTTGGTTACCATCAGCAATGCTGATTGACTTAGCATACTGGGCTACAAAGAAGAACAAGCACTCACTGATACTATTCGGTGGTGTGTTGTGTGGAATGTCACTGCCATTGTTCAACATGGTAAATCTAATTACCGTGGCTGATCCATTGGAGACTGCTTTCAAATATCCAAGACCAACATTGCCTCCATACATGACTCCAATAGAACCCCAAGTGGGCAAGTTCTATAACAGTCCAGTTGCACTCGGTGCAGGCGCAGGCGCTGTATTATCAGTAACCTTTGCCGCTCTGGGATGTAAGCTGAATACG**TGGACGTACAGATGGATGGC** |
| P5-P7 16S rRNA standard control | TGTAAAACGACGGCCAGTGAATTCGAGCTCGGTACCTCGCGAATGCATCTAGATCGTATCACCGTTTGTGTGAA**AATGATACGGCGACCACCGAGAT**CAACGAACTGAACTGGCAGACTATCCCGCCGGGAATGGTGATTACCGACGAAAACGGCAAGAAAAAGCAGTCTTACTTCCATGATTTCTTTAATTATGCCGGGATCCATCGCAGCGTAATG**TCGTATGCCGTCTTCTGCTTG**AGAGTTTGATCATGGCTCAGGGTGAACGCTGGCGGCGTGCCTAAGACATGCAAGTCGTGCGGGCCGCGGGGTTTTACTCCGTGGTCAGCGGCGGACGGGTGAATAACGCGTGGGTGACCTACCCGGAAGAGGGGGACAACCCGGGGAAACTCGGGCTAATCCCCCATGTGGACCCGCCCCTTGGGGTGTGTCCAAAGGGCTTTGCCCGCTTCCGGATGGGCCCGCGTCCCATCAGCTAGTTGGTGGGGTAATGGCCCACCAAGGCGACGACGGGTAGCCGGCCTGAGAGGGTGGCCGGCCACAGGGGCACTGAGACACGGGCCCCACTCCTACGGGAGGCAGCAGTTAGGAATCTTCCGCAATGGGCGCAAGCCTGACGGAGCGACGCCGCTTGGAGGAAGAAGCCCTTCGGGGTGTAAACTCCTGAACCCGGGACGAAACCCCCGACGAGGGGACTGACGGTACCGGGGTAATAGCGCCGGCCAACTCCGTGCCAGCAGCCGCGGTAATACGGAGGGCGCGAGCGTTACCCGGATTCACTGGGCGTAAAGGGCGTGTAGGCGGCCTGGGGCGTCCCATGTGAAAGACCACGGCTCAACCGTGGGGGAGCGTGGGATACGCTCAGGCTAGACGGTGGGAGAGGGTGGTGGAATTCCCGGAGTAGCGGTGAAATGCGCAGATACCGGGAGGAACGCCGATGGCGAAGGCAGCCACCTGGTCCACCCGTGACGCTGAGGCGCGAAAGCGTGGGGAGCAAACCGGATTAGATACCCGGGTAGTCCACGCCCTAAACGATGCGCGCTAGGTCTCTGGGTCTCCTGGGGGCCGAAGCTAACGCGTTAAGCGCGCCGCCTGGGGAGTACGGCCGCAAGGCTGAAACTCAAAGGAATTGACGGGGGCCCGCACAAGCGGTGGAGCATGTGGTTTAATTCGAAGCAACGCGAAGAACCTTACCAGGCCTTGACATGCTAGGGAACCCGGGTGAAAGCCTGGGGTGCCCCGCGAGGGGAGCCCTAGCACAGGTGCTGCATGGCCGTCGTCAGCTCGTGCCGTGAGGTGTTGGGTTAAGTCCCGCAACGAGCGCAACCCCCGCCGTTAGTTGCCAGCGGGTAGGCCGGGCACTCTAACGGGACTGCCCGCGAAAGCGGGAGGAAGGAGGGGACGACGTCTGGTCAGCATGGCCCTTACGGCCTGGGCGACACACGTGCTACAATGCCCACTACAAAGCGAGGCCACCCGGCAACGGGGAGCTAATCGCAAAAAGGTGGGCCCAGTTCGGATTGGGGTCTGCAACCCGACCCCATGAAGCCGGAATCGCTAGTAATCGCGGATCAGCCATGCCGCGGTGAATACGTTCCCGGGCCTTGTACACACCGCCCGTCACGCCATGGGAGCGGGCTCTACCCGAAGTCGCCGGGAGCCTACGGGCAGGCGCCGAGGGTAGGGCCCGTGACTGGGGCGAAGTCGTAACAAGGTAATCGGATCCCGGGCCCGTCGACTGCAGAGGCCTGCATGCAAGCTTGGCGTAATCATGGTCATAGCTGTTTCCTG |

|  |  | Comammox *Nitrospira amoA*  **Table S5.** Gene copies for both *amoA* and 16S rRNA gene targeted qPCRs for each sample. Copies are expressed per ng of extracted DNA. | | AOB *amoA* | | AOA *amoA* | | Bacterial  16S rRNA gene | | Thaumarcheotal  16S rRNA gene | |
| --- | --- | --- | --- | --- | --- | --- | --- | --- | --- | --- | --- |
| Sample ID | Type | Gene copies per ng gDNA | SD | Gene copies per ng gDNA | SD | Gene copies per ng gDNA | SD | Gene copies per ng gDNA | SD | Gene copies per ng gDNA | SD |
| FW-01 | Freshwater | 2679.1 | 104.2 | 2.3 | 0.2 | 48.0 | 3.4 | 357650.7 | 48439.3 | 337.9 | 35.8 |
| FW-02 | Freshwater | 1260.1 | 113.1 | 23.5 | 1.2 | 4.1 | 0.2 | 300566.0 | 46456.8 | 1.5 | 0.6 |
| FW-03 | Freshwater | 3328.1 | 130.7 | 3.2 | 1.2 | 73.7 | 1.8 | 259100.2 | 24032.9 | 652.1 | 25.4 |
| FW-04 | Freshwater | 3597.9 | 244.1 | 18.4 | 4.4 | 202.1 | 2.8 | 292563.9 | 41634.0 | 250.3 | 16.5 |
| FW-05 | Freshwater | 1192.6 | 13.9 | 0.8 | 0.1 | 78.2 | 1.8 | 150118.1 | 5238.3 | 356.4 | 16.9 |
| FW-06 | Freshwater | 6682.5 | 794.2 | 5.5 | 0.6 | 1062.7 | 225.2 | 277426.1 | 12601.3 | 830.7 | 0.1 |
| FW-07 | Freshwater | 1396.3 | 84.2 | 46.3 | 8.4 | 2.6 | 1.0 | 582230.8 | 97091.0 | 26.5 | 12.8 |
| FW-08 | Freshwater | 2351.5 | 4.1 | 1.7 | 0.4 | 1.6 | 0.7 | 378127.9 | 37235.2 | 10.6 | 5.3 |
| FW-09 | Freshwater | 1730.8 | 223.3 | 6.1 | 2.7 | 65.8 | 12.2 | 375128.7 | 15513.8 | 816.5 | 125.5 |
| FW-10 | Freshwater | 596.2 | 28.9 | 0.4 | 0.4 | 28.3 | 9.2 | 436530.5 | 69169.3 | 30.5 | 12.0 |
| FW-11 | Freshwater | 1235.2 | 159.7 | 0.9 | 0.4 | 0.7 | 0.1 | 180345.5 | 2670.1 | 42.3 | 5.6 |
| FW-12 | Freshwater | 2453.8 | 98.4 | 1.4 | 0.1 | 13453.9 | 1552.3 | 381424.0 | 81179.0 | 21162.7 | 4009.6 |
| FW-13 | Freshwater | 3761.0 | 2.4 | 12.3 | 3.3 | 17.1 | 0.8 | 238815.4 | 1040.4 | 99.7 | 14.1 |
| FW-14 | Freshwater | 5010.0 | 1046.4 | 1.7 | 0.2 | 2368.9 | 210.1 | 240915.5 | 44783.9 | 1792.9 | 210.7 |
| FW-15 | Freshwater | 1455.3 | 131.4 | 0.5 | 0.1 | 1976.5 | 279.9 | 334006.2 | 267705.7 | 2488.2 | 370.2 |
| FW-16 | Freshwater | 1445.2 | 216.3 | 1.6 | 0.1 | 15.6 | 1.6 | 354042.2 | 8000.5 | 146.9 | 9.5 |
| FW-17 | Freshwater | 2364.1 | 447.8 | 2.2 | 0.2 | 256.3 | 33.6 | 612831.7 | 136570.8 | 405.7 | 102.7 |
| FW-18 | Freshwater | 842.5 | 61.6 | 0.5 | 0.1 | 1.8 | 0.1 | 106739.5 | 19987.6 | 2.0 | 0.2 |
| FW-19 | Freshwater | 645.3 | 83.0 | 0.6 | 0.2 | 70.4 | 12.9 | 238073.3 | 70781.2 | 356.5 | 95.3 |
| FW-20 | Freshwater | 960.3 | 58.1 | 109.0 | 10.4 | BDL | - | 100728.4 | 3753.2 | 0.5 | 0.0 |
| FW-21 | Freshwater | 545.3 | 21.1 | 1.7 | 0.1 | 358.6 | 11.3 | 386154.4 | 109426.1 | 524.1 | 16.6 |
| FW-22 | Freshwater | 1179.2 | 130.8 | 138.1 | 31.9 | 8.8 | 2.3 | 602839.7 | 28901.7 | 474.5 | 31.6 |
| FW-23 | Freshwater | 4368.9 | 77.4 | 689.0 | 196.6 | 116.7 | 6.4 | 1210216.3 | 237183.9 | 202.6 | 36.6 |
| FW-24 | Freshwater | 1765.9 | 227.4 | 12.1 | 2.2 | 2019.4 | 223.8 | 1307997.8 | 1389547.8 | 2093.8 | 172.0 |
| FW-25 | Freshwater | 461.4 | 36.7 | 2.1 | 0.4 | 646.3 | 9.3 | 101620.5 | 23826.8 | 1310.2 | 178.9 |
| FW-26 | Freshwater | 2238.6 | 476.5 | 8.3 | 0.2 | 165.9 | 32.3 | 566035.2 | 153888.7 | 1056.8 | 32.0 |
| FW-27 | Freshwater | 2786.2 | 430.2 | 3.5 | 0.0 | 178.4 | 46.7 | 463383.5 | 30854.2 | 437.5 | 88.3 |
| FW-28 | Freshwater | 884.1 | 130.8 | 2.0 | 0.3 | 2359.7 | 357.4 | 149997.0 | 7441.8 | 1968.6 | 584.8 |
| FW-29 | Freshwater | 4973.7 | 957.6 | 4.9 | 0.6 | 1134.3 | 217.1 | 545152.5 | 51591.8 | 1805.4 | 263.4 |
| FW-30 | Freshwater | 2926.2 | 398.5 | 1.4 | 0.2 | 462.2 | 4.9 | 267397.7 | 35865.0 | 512.1 | 55.5 |
| FW-31 | Freshwater | 1674.7 | 118.6 | 2.5 | 0.6 | 2575.8 | 343.2 | 121920.8 | 14029.9 | 743.9 | 65.6 |
| FW-32 | Freshwater | 1570.9 | 0.5 | 1.4 | 0.1 | 138.5 | 3.6 | 311943.9 | 22899.5 | 264.6 | 35.4 |
| FW-33 | Freshwater | 1138.0 | 112.3 | 5.9 | 0.1 | BDL | - | 387222.8 | 149070.7 | 2.5 | 0.3 |
| FW-34 | Freshwater | 34.6 | 0.0 | 68.3 | 10.4 | BDL | - | 176799.5 | 7174.0 | 2.2 | 0.2 |
| FW-35 | Freshwater | 1467.0 | 301.3 | 4.5 | 0.4 | 1.2 | 0.0 | 957348.9 | 799163.3 | 10.3 | 4.4 |
| FW-36 | Freshwater | 4729.0 | 383.1 | 3.9 | 0.9 | 9922.1 | 542.0 | 432699.9 | 68186.8 | 14471.5 | 2056.0 |
| FW-37 | Freshwater | 3233.5 | 296.8 | 15.3 | 1.4 | 779.2 | 62.0 | 119497.4 | 14233.0 | 338.1 | 4.8 |
| FW-38 | Freshwater | 1594.3 | 179.4 | 0.8 | 0.5 | 2.1 | 0.9 | 136777.2 | 23155.9 | 40.0 | 4.9 |
| SW-01 | Saltwater | BDL | - | 218.5 | 8.4 | 631.1 | 81.2 | 204763.7 | 45638.6 | 4830.5 | 364.3 |
| SW-02 | Saltwater | BDL | - | 348.4 | 14.2 | 354.3 | 42.3 | 166481.8 | 36453.6 | 6319.1 | 1725.6 |
| SW-03 | Saltwater | BDL | - | 2.3 | 0.9 | 69.0 | 16.3 | 137252.7 | 29006.7 | 2367.6 | 57.8 |
| SW-04 | Saltwater | BDL | - | 107.1 | 6.4 | 194.3 | 15.2 | 240120.1 | 14018.5 | 3476.7 | 411.5 |
| SW-05 | Saltwater | BDL | - | 261.3 | 12.8 | 29.9 | 2.2 | 393031.5 | 238249.5 | 896.2 | 233.9 |
| SW-06 | Saltwater | BDL | - | 264.3 | 29.2 | 59.2 | 2.4 | 286943.4 | 18283.7 | 165.9 | 12.5 |
| SW-07 | Saltwater | BDL | - | 393.7 | 96.9 | 596.8 | 8.5 | 240783.8 | 17034.1 | 8470.4 | 516.0 |
| SW-08 | Saltwater | BDL | - | 18.5 | 3.3 | 28.4 | 5.8 | 263155.1 | 4273.9 | 480.5 | 69.6 |

**Table S6.** Summary of amplicon read counts before, during, and after final filtration of *amoA* amplicon sequence data.

| **Read counts** | **Original ASV table** | **Post R script filtering** | **ASV table post blast filtering (final)** | | **% reads/ASVs after filtering** |
| --- | --- | --- | --- | --- | --- |
| ***Average read length (bp)*** | ***350.8*** | ***349.5*** | ***380.5*** | ***n/a*** | |
| Total number of ASVs | 1535 | 976 | 248 | 16.2 | |
| Total read count | 150430 | 149601 | 110464 | 73.4 | |
| RBC Positive Control | 5813 | 5813 | 5813 | 100.0 | |
| PCR Negative Control | 8 | 8 | 8 | 100.0 | |
| Aq-C1 | 1367 | 1362 | 1360 | 99.5 | |
| Aq-C2 | 1459 | 1459 | 1457 | 99.9 | |
| FW-01 | 3458 | 3429 | 3164 | 91.5 | |
| FW-02 | 4243 | 4237 | 3905 | 92.0 | |
| FW-03 | 1940 | 1895 | 1359 | 70.1 | |
| FW-04 | 821 | 808 | 575 | 70.0 | |
| FW-05 | 3755 | 3698 | 3595 | 95.7 | |
| FW-06 | 3776 | 3697 | 3561 | 94.3 | |
| FW-07 | 3806 | 3802 | 2070 | 54.4 | |
| FW-08 | 4328 | 4326 | 3000 | 69.3 | |
| FW-09 | 4594 | 4576 | 1871 | 40.7 | |
| FW-10 | 1936 | 1855 | 700 | 36.2 | |
| FW-11 | 2856 | 2829 | 2397 | 83.9 | |
| FW-12 | 5007 | 5001 | 4598 | 91.8 | |
| FW-13 | 4396 | 4358 | 3008 | 68.4 | |
| FW-14 | 4101 | 4077 | 3975 | 96.9 | |
| FW-15 | 3604 | 3560 | 3339 | 92.6 | |
| FW-16 | 5735 | 5735 | 2945 | 51.4 | |
| FW-17 | 3108 | 3101 | 2243 | 72.2 | |
| FW-18 | 6508 | 6492 | 5319 | 81.7 | |
| FW-19 | 3691 | 3673 | 2078 | 56.3 | |
| FW-20 | 5823 | 5817 | 5513 | 94.7 | |
| FW-21 | 4711 | 4679 | 3588 | 76.2 | |
| FW-22 | 5039 | 5029 | 3615 | 71.7 | |
| FW-23 | 3762 | 3762 | 1451 | 38.6 | |
| FW-24 | 3279 | 3273 | 1712 | 52.2 | |
| FW-25 | 968 | 924 | 757 | 78.2 | |
| FW-26 | 3600 | 3569 | 2672 | 74.2 | |
| FW-27 | 6239 | 6203 | 4391 | 70.4 | |
| FW-28 | 3832 | 3821 | 2882 | 75.2 | |
| FW-29 | 4527 | 4508 | 3977 | 87.9 | |
| FW-30 | 2803 | 2803 | 2673 | 95.4 | |
| FW-31 | 3087 | 3085 | 2744 | 88.9 | |
| FW-32 | 2818 | 2809 | 2144 | 76.1 | |
| FW-33 | 4258 | 4256 | 2142 | 50.3 | |
| FW-34 | 1681 | 1624 | 54 | 3.2 | |
| FW-35 | 6694 | 6674 | 2470 | 36.9 | |
| FW-36 | 3260 | 3247 | 2512 | 77.1 | |
| FW-37 | 4292 | 4280 | 3421 | 79.7 | |
| FW-38 | 5268 | 5268 | 5227 | 99.2 | |

*Note – target sequence for amplification of comammox amoA gene is 381 bp in length.*

**Table S7.** GenBank identifiers and description for AmoA sequences previously

| **AmoA ID** | **GenBank accession** | **Species/metagenome association or description** |
| --- | --- | --- |
| AmoA-01 | [QEP54817.1](https://www.ncbi.nlm.nih.gov/protein/QEP54817.1?report=genbank&log$=prottop&blast_rank=1&RID=2J8NBH2201R) | ammonia monooxygenase subunit A, partial [uncultured bacterium] pasture soil |
| **AmoA-02** | [VWF18674.1](https://www.ncbi.nlm.nih.gov/protein/VWF18674.1?report=genbank&log$=prottop&blast_rank=2&RID=2J8NBH2201R) | Guelph RBC Group D *Nitrospira* sp. MAG |
| **AmoA-03** | [VWF18671.1](https://www.ncbi.nlm.nih.gov/protein/VWF18671.1?report=genbank&log$=prottop&blast_rank=3&RID=2J8NBH2201R) | Guelph RBC Group A *Nitrospira* sp. MAG |
| **AmoA-04** | [NJL18594.1](https://www.ncbi.nlm.nih.gov/protein/NJL18594.1?report=genbank&log$=prottop&blast_rank=2&RID=2J8NBH2201R) | Guelph RBC Group C *Nitrospira* sp. MAG |
| AmoA-05 | [QIH54442.1](https://www.ncbi.nlm.nih.gov/protein/QIH54442.1?report=genbank&log$=prottop&blast_rank=1&RID=2J8NBH2201R) | ammonia monooxygenase, partial [*Nitrospira* sp. enrichment culture] fast sand filter |
| AmoA-12 | [ASO97026.1](https://www.ncbi.nlm.nih.gov/protein/ASO97026.1?report=genbank&log$=prottop&blast_rank=1&RID=2J8NBH2201R) | ammonia monooxygenase, partial [uncultured bacterium] drinking water treatment plant |
| AmoA-15 | [QXU65026.1](https://www.ncbi.nlm.nih.gov/protein/QXU65026.1?report=genbank&log$=prottop&blast_rank=1&RID=2J8NBH2201R) | ammonia monooxygenase subunit A, partial [uncultured *Nitrospira* sp.] tidal flat wetland estuary |
| AmoA-17 | [QXU65031.1](https://www.ncbi.nlm.nih.gov/protein/QXU65031.1?report=genbank&log$=prottop&blast_rank=1&RID=2J8NBH2201R) | ammonia monooxygenase subunit A, partial [uncultured *Nitrospira* sp.] tidal flat wetland estuary |
| AmoA-18 | [QXU65075.1](https://www.ncbi.nlm.nih.gov/protein/QXU65075.1?report=genbank&log$=prottop&blast_rank=1&RID=2J8NBH2201R) | ammonia monooxygenase subunit A, partial [uncultured *Nitrospira* sp.] tidal flat wetland estuary |
| AmoA-19 | [QXU65075.1](https://www.ncbi.nlm.nih.gov/protein/QXU65075.1?report=genbank&log$=prottop&blast_rank=1&RID=2J8NBH2201R) | ammonia monooxygenase subunit A, partial [uncultured *Nitrospira* sp.] tidal flat wetland estuary |
| AmoA-20 | [QXU65075.1](https://www.ncbi.nlm.nih.gov/protein/QXU65075.1?report=genbank&log$=prottop&blast_rank=1&RID=2J8NBH2201R) | ammonia monooxygenase subunit A, partial [uncultured *Nitrospira* sp.] tidal flat wetland estuary |
| AmoA-26 | [QXU65099.1](https://www.ncbi.nlm.nih.gov/protein/QXU65099.1?report=genbank&log$=prottop&blast_rank=1&RID=2J8NBH2201R) | ammonia monooxygenase subunit A, partial [uncultured *Nitrospira* sp.] tidal flat wetland estuary |
| AmoA-29 | [QXU65161.1](https://www.ncbi.nlm.nih.gov/protein/QXU65161.1?report=genbank&log$=prottop&blast_rank=1&RID=2J8NBH2201R) | ammonia monooxygenase subunit A, partial [uncultured *Nitrospira* sp.] tidal flat wetland estuary |
| **AmoA-30** | [WP_090742150.1](https://www.ncbi.nlm.nih.gov/protein/WP_090742150.1?report=genbank&log$=prottop&blast_rank=9&RID=2J8NBH2201R) | *Ca.* Nitrospira nitrosa |
| **AmoA-31** | [LVWU01000285.1](https://www.ncbi.nlm.nih.gov/nuccore/LVWU01000285.1) | *Nitrospira* sp. HN-bin3 MAG |
| AmoA-32 | [MBS0156953.1](https://www.ncbi.nlm.nih.gov/protein/MBS0156953.1?report=genbank&log$=prottop&blast_rank=1&RID=2J8NBH2201R) | methane/ammonia monooxygenase subunit A [*Nitrospira* sp.] activated sludge metagenome |
| AmoA-33 | [QXU65083.1](https://www.ncbi.nlm.nih.gov/protein/QXU65083.1?report=genbank&log$=prottop&blast_rank=1&RID=2J8NBH2201R) | ammonia monooxygenase subunit A, partial [uncultured *Nitrospira* sp.] tidal flat wetland estuary |
| AmoA-34 | [QXU65170.1](https://www.ncbi.nlm.nih.gov/protein/QXU65170.1?report=genbank&log$=prottop&blast_rank=1&RID=2J8NBH2201R) | ammonia monooxygenase subunit A, partial [uncultured *Nitrospira* sp.] tidal flat wetland estuary |
| AmoA-35 | [QXU65063.1](https://www.ncbi.nlm.nih.gov/protein/QXU65063.1?report=genbank&log$=prottop&blast_rank=1&RID=2J8NBH2201R) | ammonia monooxygenase subunit A, partial [uncultured *Nitrospira* sp.] tidal flat wetland estuary |
| AmoA-36 | [QXU65144.1](https://www.ncbi.nlm.nih.gov/protein/QXU65144.1?report=genbank&log$=prottop&blast_rank=1&RID=2J8NBH2201R) | ammonia monooxygenase subunit A, partial [uncultured *Nitrospira* sp.] tidal flat wetland estuary |
| AmoA-37 | [QXU65138.1](https://www.ncbi.nlm.nih.gov/protein/QXU65138.1?report=genbank&log$=prottop&blast_rank=1&RID=2J8NBH2201R) | ammonia monooxygenase subunit A, partial [uncultured *Nitrospira* sp.] tidal flat wetland estuary |
| AmoA-38 | [QXE97099.1](https://www.ncbi.nlm.nih.gov/protein/QXE97099.1?report=genbank&log$=prottop&blast_rank=1&RID=2J8NBH2201R) | ammonia monooxygenase subunit A, partial [uncultured *Nitrospira* sp.] agricultural soil |
| AmoA-41 | [QXE97092.1](https://www.ncbi.nlm.nih.gov/protein/QXE97092.1?report=genbank&log$=prottop&blast_rank=1&RID=2J8NBH2201R) | ammonia monooxygenase subunit A, partial [uncultured *Nitrospira* sp.] agricultural soil |
| AmoA-42 | [QPD98999.1](https://www.ncbi.nlm.nih.gov/protein/QPD98999.1?report=genbank&log$=prottop&blast_rank=1&RID=2J8NBH2201R) | ammonia monooxygenase subunit A, partial [uncultured bacterium] forest soil |
| AmoA-43 | [QXE97180.1](https://www.ncbi.nlm.nih.gov/protein/QXE97180.1?report=genbank&log$=prottop&blast_rank=1&RID=2J8NBH2201R) | ammonia monooxygenase subunit A, partial [uncultured *Nitrospira* sp.] agricultural soil |
| AmoA-45 | [ASO97339.1](https://www.ncbi.nlm.nih.gov/protein/ASO97339.1?report=genbank&log$=prottop&blast_rank=1&RID=2J8NBH2201R) | ammonia monooxygenase, partial [uncultured bacterium] rice paddy soil |
| AmoA-46 | [QEP54822.1](https://www.ncbi.nlm.nih.gov/protein/QEP54822.1?report=genbank&log$=prottop&blast_rank=1&RID=2J8NBH2201R) | ammonia monooxygenase subunit A, partial [uncultured bacterium] vegetable soil |
| AmoA-49 | [QXE96988.1](https://www.ncbi.nlm.nih.gov/protein/QXE96988.1?report=genbank&log$=prottop&blast_rank=1&RID=2J8NBH2201R) | ammonia monooxygenase subunit A, partial [uncultured *Nitrospira* sp.] agricultural soil |
| **AmoA-52** | OQW57195.1 | *Nitrospira* sp. ST-bin4 MAG |
| AmoA-54 | [QEP54815.1](https://www.ncbi.nlm.nih.gov/protein/QEP54815.1?report=genbank&log$=prottop&blast_rank=1&RID=2NC2PZXX013) | ammonia monooxygenase subunit A, partial [uncultured bacterium] pasture soil |
| AmoA-55 | [BBI25072.1](https://www.ncbi.nlm.nih.gov/protein/BBI25072.1?report=genbank&log$=prottop&blast_rank=1&RID=2NC2PZXX013) | ammonia monooxygenase subunit A, partial [uncultured *Nitrospira* sp.] nitrifying granule |
| AmoA-57 | [QOD39640.1](https://www.ncbi.nlm.nih.gov/protein/QOD39640.1?report=genbank&log$=prottop&blast_rank=1&RID=2NC2PZXX013) | ammonia monooxygenase subunit A, partial [uncultured *Nitrospira* sp.] tidal flat wetland estuary |
| AmoA-59 | [QXU65066.1](https://www.ncbi.nlm.nih.gov/protein/QXU65066.1?report=genbank&log$=prottop&blast_rank=1&RID=2NC2PZXX013) | ammonia monooxygenase subunit A, partial [uncultured *Nitrospira* sp.] tidal flat wetland estuary |
| AmoA-63 | [QXU65054.1](https://www.ncbi.nlm.nih.gov/protein/QXU65054.1?report=genbank&log$=prottop&blast_rank=1&RID=2NC2PZXX013) | ammonia monooxygenase subunit A, partial [uncultured *Nitrospira* sp.] tidal flat wetland estuary |
| AmoA-64 | [ASO96973.1](https://www.ncbi.nlm.nih.gov/protein/ASO96973.1?report=genbank&log$=prottop&blast_rank=1&RID=2NC2PZXX013) | ammonia monooxygenase, partial [uncultured bacterium] groundwater well |
| **AmoA-65** | THJ16210.1 | *Nitrospira* sp. CG24B MAG |
| AmoA-67 | [ASO97348.1](https://www.ncbi.nlm.nih.gov/protein/ASO97348.1?report=genbank&log$=prottop&blast_rank=1&RID=2NC2PZXX013) | ammonia monooxygenase, partial [uncultured bacterium] recirculating aquarium filter |
| **AmoA-68** | CUS38627.1 | *Ca.* Nitrospira nitrificans |
| **AmoA-69** | [VWF18676.1](https://www.ncbi.nlm.nih.gov/protein/VWF18676.1?report=genbank&log$=prottop&blast_rank=4&RID=2NC2PZXX013) | Guelph RBC Group G *Nitrospira* sp. MAG |
| AmoA-70 | [QXU65036.1](https://www.ncbi.nlm.nih.gov/protein/QXU65036.1?report=genbank&log$=prottop&blast_rank=1&RID=2NC2PZXX013) | ammonia monooxygenase subunit A, partial [uncultured *Nitrospira* sp.] tidal flat wetland estuary |
| AmoA-71 | [QXU65169.1](https://www.ncbi.nlm.nih.gov/protein/QXU65169.1?report=genbank&log$=prottop&blast_rank=1&RID=2NC2PZXX013) | ammonia monooxygenase subunit A, partial [uncultured *Nitrospira* sp.] tidal flat wetland estuary |
| AmoA-73 | [UBY00756.1](https://www.ncbi.nlm.nih.gov/protein/UBY00756.1?report=genbank&log$=prottop&blast_rank=1&RID=2NC2PZXX013) | ammonia monooxygenase, partial [uncultured *Nitrospira* sp.] tributary sediment |

**
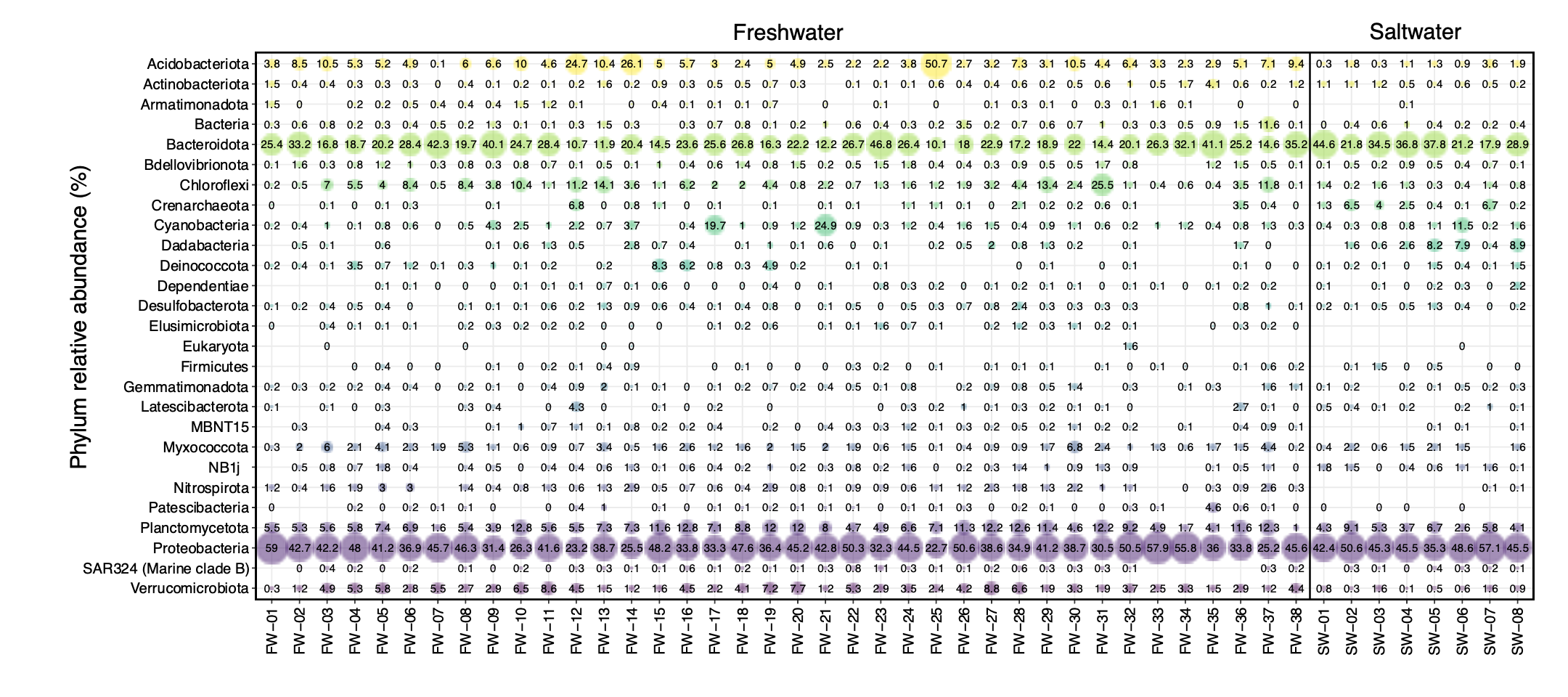
**

**Figure S1** The relative abundance plot of 16S rRNA gene profiles of aquarium biofilter samples at the phylum level (C) shows taxonomy assigned using the SILVA 138 SSU database. Size of the bubble and the number represent the relative abundance (RA) of each phylum within a biofilter sample. Only phyla present in at least one biofilter at a relative abundance of <1% are shown.

**
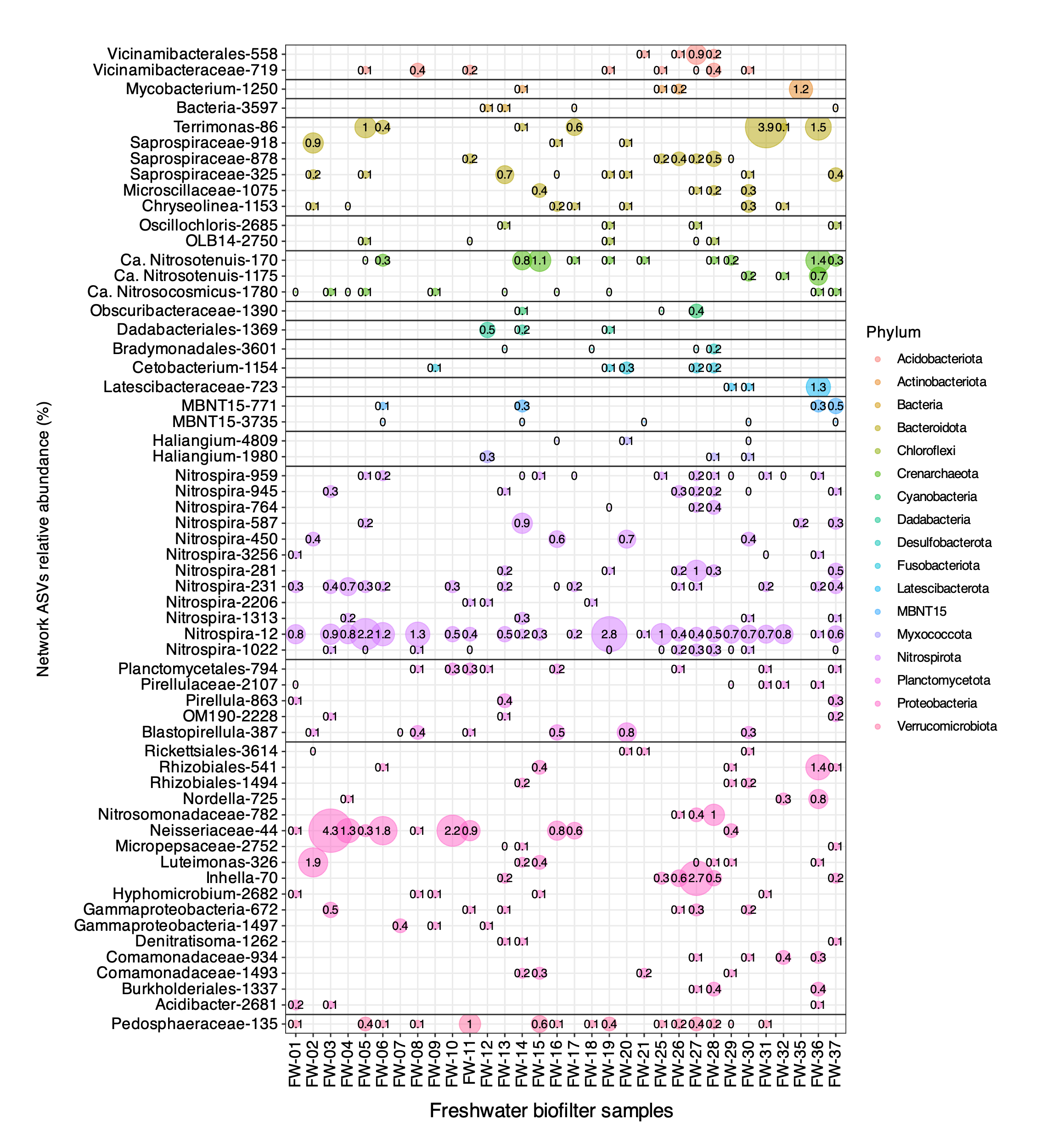
**

**Figure S2** Relative abundance plot showing the subset of ASVs presented in the FlashWeave network analysis (Fig. 5), across all freshwater aquarium biofilter samples. Relative abundance values are shown as relative to the entire microbial community, without filtering any ASVs, with the number and bubble size representing the relative abundance of each ASV within each sample.
